# The *Brassica rapa FLC* homologue *FLC2* is a key regulator of flowering time, identified through transcriptional co-expression networks

**DOI:** 10.1093/jxb/ert264

**Published:** 2013-09-27

**Authors:** Dong Xiao, Jian J. Zhao, Xi L. Hou, Ram K. Basnet, Dunia P.D. Carpio, Ning W. Zhang, Johan Bucher, Ke Lin, Feng Cheng, Xiao W. Wang, Guusje Bonnema

**Affiliations:** ^1^Wageningen UR Plant Breeding, PO Box 386, AJ 6700 Wageningen, The Netherlands; ^2^State Key Laboratory of Crop Genetics and Germplasm Enhancement, Horticultural College, Nanjing Agricultural University, Nanjing, 210095 Jiangsu, China; ^3^Horticultural College, Hebei Agricultural University, 071001 Baoding, China; ^4^Center for Biosystems Genomics, Wageningen University, AJ 6700 Wageningen, The Netherlands; ^5^Institute of Developmental Genetics, Heinrich-Heine University, D-40225 Düsseldorf, Germany; ^6^Institute of Vegetables and Flowers, Chinese Academy of Agricultural Sciences, 100081 Beijing, China

**Keywords:** *Brassica rapa*, candidate gene mapping, expression quantitative trait loci (eQTL), *FLOWERING LOCUS C (FLC)*., flowering time, gene expression networks, genome triplication.

## Abstract

The role of many genes and interactions among genes involved in flowering time have been studied extensively in *Arabidopsis*, and the purpose of this study was to investigate how effectively results obtained with the model species *Arabidopsis* can be applied to the *Brassicacea* with often larger and more complex genomes. *Brassica rapa* represents a very close relative, with its triplicated genome, with subgenomes having evolved by genome fractionation. The question of whether this genome fractionation is a random process, or whether specific genes are preferentially retained, such as flowering time (Ft) genes that play a role in the extreme morphological variation within the *B. rapa* species (displayed by the diverse morphotypes), is addressed. Data are presented showing that indeed Ft genes are preferentially retained, so the next intriguing question is whether these different orthologues of *Arabidopsis* Ft genes play similar roles compared with *Arabidopsis*, and what is the role of these different orthologues in *B. rapa*. Using a genetical–genomics approach, co-location of flowering quantitative trait loci (QTLs) and expression QTLs (eQTLs) resulted in identification of candidate genes for flowering QTLs and visualization of co-expression networks of Ft genes and flowering time. A major flowering QTL on A02 at the *BrFLC2* locus co-localized with *cis* eQTLs for *BrFLC2*, *BrSSR1*, and *BrTCP11,* and *trans* eQTLs for the photoperiod gene *BrCO* and two paralogues of the floral integrator genes *BrSOC1* and *BrFT*. It is concluded that the *BrFLC2* Ft gene is a major regulator of flowering time in the studied doubled haploid population.

## Introduction

In flowering plants, the change from vegetative to reproductive development is a major transition that is sensitive to various seasonal climatic signals (Andres and Coupland, 2012). Controlling the timing of this transition is especially important in crop plants as it determines the geographical range where the crop can be cultivated and ensures high agricultural productivity. Genetic factors that control flowering time are well studied in the model plant *Arabidopsis*. Control of flowering in *Arabidopsis* is the result of an interaction of environmental and physiological factors in different pathways: the vernalization pathway, photoperiod/circadian clock pathway, autonomous pathway, ageing pathway, ambient temperature pathway, and gibberellin pathway (Roux *et al.*, 2006; Alonso-Blanco *et al.*, 2009; Fornara *et al.*, 2010). In total, >300 genes have been implicated in the control of flowering in *Arabidopsis*, and the key regulatory components involved in different pathways and their interactions are well summarized (Fornara *et al.*, 2010).

Polyploidy [whole-genome duplication (WGD)] has played an important role in evolution and genetic diversity of angiosperm genomes (De Bodt *et al.*, 2005; Flagel and Wendel, 2009). WGD events are generally followed by changes in gene expression and widespread gene loss (Sankoff *et al.*, 2010). The *Brassica* genus is closely related to the model species *Arabidopsis thaliana*, both members of the *Brassicaceae* family. Six *Brassica* species are cultivated worldwide: three diploids, *B. rapa* (AA, 2*n*=20), *B. nigra* (BB, 2*n*=16), and *B. oleracea* (CC, 2*n*=18); and three amphidiploids, *B. juncea* (AABB, 2*n*=36), *B. napus* (AACC, 2*n*=38), and *B. carinata* (BBCC, 2*n*=34). Comparative genetic and physical mapping and genome sequencing studies have confirmed the syntenic relationship between *Arabidopsis* and the triplicated genome of *B. rapa*, with subgenomes having evolved by genome fractionation (Park *et al*., 2005; Wang *et al.*, 2011; Cheng *et al.*, 2012; Tang *et al.*, 2012).

The paleohexaploid crop *B. rapa* displays extreme morphological diversity, and includes leafy vegetables, turnips, and oil types that all differ based on which organs are consumed (Zhao *et al.*, 2005; Bonnema *et al.*, 2011). Flowering time is an important developmental trait, and wide variation exist both between and within *B. rapa* morphotypes. Previous studies on flowering time regulation in *Brassica* have identified some major flowering candidate genes such as *FLOWERING LOCUS C* (*FLC*), *CONSTANS* (*CO*), and *FLOWERING LOCUS T* (*FT*). In *Arabidopsis*, the *FLC* MADS-box gene is a target of the autonomous flowering time and vernalization pathways that acts in a dosage-dependent manner to repress flowering (Michaels and Amasino, 1999). No null allele or copy number variation of *FLC* has been identified in *Arabidopsis* ecotypes (Michaels *et al.*, 2003). Four *FLC* paralogues (*FLC1*, *FLC2*, *FLC3*, and *FLC5*) each in *B. rapa* (Schranz *et al.*, 2002) and in *B. oleracea* (Okazaki *et al.*, 2007) have been cloned. Co-localization of *FLC* paralogues with quantitative trait loci (QTLs) for flowering time in *B. rapa* (Lou *et al.*, 2007; Yuan *et al.*, 2009) and study of *BrFLC* expression in Chinese cabbage (Kim *et al.*, 2007) and in *B. napus* (Tadege *et al.*, 2001; Zou *et al.*, 2012) indicates that the *Brassica FLC* genes act similarly to *Arabidopsis FLC*. Co-localization of a major QTL for both flowering time and vernalization response with the *BrFLC2* locus and correlation between *BrFLC2* transcript levels, vernalization treatments, and flowering time also suggested that the role of *BrFLC2* in *B. rapa* is comparable with that of *FLC* in *Arabidopsis* (Zhao *et al.*, 2010). However, in another study using a collection of *B. rapa* accessions and a Chinese cabbage double haploid (DH) population, a splicing site mutation in *BrFLC1* was significantly associated with flowering time (Yuan *et al.*, 2009). This illustrates that in different genetic backgrounds, different *FLC* paralogues have major effects on flowering time. In addition, six *FT* paralogues in *B. napus* have been mapped; three of them were associated with two major QTL clusters for flowering time (Wang *et al.*, 2009).

Jansen and Nap (2001) proposed the term ‘Genetical–Genomics’, in which mRNA transcript abundances can be treated as quantitative traits to map expression QTLs (eQTLs). In this way, eQTLs can be classified as *cis*-acting (if the gene and QTL co-localize) and *trans*-acting (no co-localization). Recently this approach has been widely applied in different organisms (Keurentjes *et al.*, 2007; Zhang *et al.*, 2011) to gain insight into the genetics of traits of interest. In a genetical–genomics study in *Arabidopsis*, regulatory networks of genes involved in the transition to flowering have been constructed by using eQTLs identified in a genome-wide gene expression study of a recombinant inbred line (RIL) population (Keurentjes *et al.*, 2007).

To understand the genetic architecture of flowering time by a genetical–genomics approach in *B. rapa*, a large number of genetic markers were developed based on *Arabidopsis* flowering time genes (Ft genes) that were identified in BLAST searches against the Chinese cabbage Chiifu-401 genome sequence (Wang *et al.*, 2011), and could address whether Ft genes are preferentially retained in the *B. rapa* genome. A subset of these genes were mapped in a *B. rapa* DH population (DH68) developed from a cross between a Yellow sarson (YS-143) and a Pak choi (PC-175), which is a cross reciprocal to an earlier studied population DH38 (Lou *et al.*, 2007; Zhao *et al.*, 2010). These markers connect the physical and genetic *B. rapa* maps, which is a prerequisite for genetical–genomic studies. To understand how flowering time is regulated when many Ft genes are retained in multiple copies, and to reveal the roles of these genes in the specific population and growth conditions studied here, flowering QTLs were identified in the DH68 population and leaves of young DH plants were profiled for whole-genome transcript variation using a distant pair design with the Cogenics 60-mer oligo Microarray (Trick *et al.*, 2009). A genome-wide eQTL analysis was performed and gene networks related to flowering time were constructed to explore the intra- or inter-relationship of genes involved in flowering time pathways. Finally, correlations between transcript abundance and flowering time variation, combined with genetic positional information of flowering QTLs and eQTLs allowed the Ft genes that regulate flowering time to be defined and to gain insight into the complex flowering time network in the paleohexaploid crop *B. rapa*.

## Materials and methods

### Plant materials and growth conditions

A *B. rapa* DH population DH68 of 163 DH lines was established from three F_1_ plants of a cross between Yellow sarson YS-143 (accession no. FIL500) as the female parent and Pak choi PC-175 (cultivar: Nai Bai Cai; accession no. VO2B0226) as the male parent. A set of 92 DH lines was evaluated for flowering time from September to December 2008. Seedlings were germinated on wet filter paper at 25 °C in the dark for 2 d, and then transplanted to nursery trays to be cultured in a greenhouse for 2 weeks. Plants with 4–5 true leaves were transplanted to soil (pots of Ø 17cm). Plants were grown in a heated greenhouse (18–21 °C) at Wageningen, The Netherlands. When the days were shorter than 16h, artificial light was supplied until a photoperiod (200 μmol m^–2^ s^–1^) of 16h. DH lines were planted in three blocks arranged in a randomized design, and flowering time was scored for one plant per block.

Flowering time was defined as number of days from transplanting to appearance of the first open flower.

### Development of genetic markers for genes involved in flowering time regulation

A total of 365 *Arabidopsis* genes were selected from the literature (Roux *et al.*, 2006; Brachi *et al.*, 2010; Fornara *et al.*, 2010) and databases (http://www.arabidopsis.org/) that are described as being implicated in flowering time control in *Arabidopsis*. Using the *Arabidopsis* gene sequence as query, homologous genes in *B. rapa* were identified in BLASTn searches against the annotated Chinese cabbage Chiifu genome sequence (http://brassicadb.org/brad/index.php) (Wang *et al.*, 2011) (Supplementary Table S1 available at *JXB* online). All *B. rapa* annotations matching the same *Arabidopsis* genes generated by BLASTP best hit were considered as *B. rapa* paralogues (cut-off E-value e^–5^). Gene structures were predicted by sequence comparison with the *Arabidopsis* CDS by DNASTAR Lasergene 9.0 (Lasergene, USA) (Supplementary Table S1).

For each gene/paralogue, three sets of primers were designed based on the genomic sequence of candidate genes to amplify 84–619bp fragments uniquely (Supplementary Table S2 at *JXB* online). The primers are named following the gene nomenclature system of *Brassica* ordered from left to right (genus–species–gene–name–genome–locus) (Østergaard and King, 2008). Primer pairs that gave a clear single band on agarose gel that was polymorphic between the parents of DH68 using the LightScanner scoring were profiled over the DH68 population. LightScanner is an extremely sensitive, accurate, and reliable technique for genotyping studies (http://www.biofiredx.com/MutationDiscovery/index.html) (Idaho Technology Inc., USA).

### Genetic map position of gene-targeted makers

Genomic DNA was extracted from fresh leaves of the 163 DH68 lines according to the procedure described by Beek *et al.* (1992). PCR was performed in 10 μl volumes: 2 μl of 5× PCR buffer, 0.4 μl of 2mM of dNTP, 0.5 μl of 2.5mM of both forward and reverse primers, 1 μl of 1×LC-green Plus, 0.1 μl of 1U of Phire enzyme, 1 μl of 50ng of genomic DNA, and 4.5 μl of double-distilled water. PCRs were performed on a 7300 Thermo cycler (Bio-RAD, USA). The temperature cycling protocol consists of an initial denaturation step at 98 °C for 30 s, followed by 40 cycles of denaturation at 98 °C for 10 s, annealing at 60 °C for 10 s, and an extension at 72 °C for 30 s. This PCR was followed by another denaturation step at 94 °C for 30 s, followed by cooling down to 25 °C for 30 s to facilitate heteroduplex formation. Following PCR, each product was transferred to a 96-well LightScanner^®^ System (Idaho Technology Inc., USA) for high resolution melting (HRM) analysis and covered with a mineral oil overlay. Samples were heated from 70 °C up to 96 °C with a ramp rate of 0.10 °C s^–1^. LightScanner software (Version 2.0) was used for data analysis following the protocols described in Montgomery *et al.* (2007). A saturating DNA binding dye is introduced during DNA amplification, which enables differentiation of PCR products based on their dissociation behaviour as they are subjected to increasing temperatures. Melting profiles were calibrated by internal oligonucleotide controls, and then normalized, temperature shifted, grouped. and displayed as fluorescence versus temperature plots (Montgomery *et al.*, 2007).

A total of 125 flowering time gene-targeted (FTGT) markers together with AFLP (amplified fragment length polymorphism), SSR (simple sequence repeat), and InDel markers were used to construct the genetic map using the program Joinmap 4.0 (Kyazma, Wageningen, The Netherlands) (http://www.kyazma.nl). The 10 linkage groups of *B. rapa* were named A01–A10 corresponding to the reference map (http://brassicadb.org/brad/geneticMarker.php?chr=ALL). To investigate the quality of the genetic map, the marker order of the genetic and physical Chiifu-401 map was compared by placing the genetically mapped candidate Ft genes on the physical map using annotated gene position number by blasting the *Brassica* database (http://brassicadb.org/brad/searchAll.php) (Supplementary Table S1 at *JXB* online).

### Flowering time QTL analysis

Flowering time QTLs (flowering QTLs) were identified by composite interval mapping using the software MAPQTL 5.0 (Van Ooijen, 2004). A permutation test was applied to each data set (1000 repetitions) to decide the LOD (logarithm of odds) thresholds (*P*=0.05). In this study, a LOD value of 3.0 was used as a significant threshold. Flowering QTLs were graphically displayed using Map chart 2.2 (Voorrips, 2002).

### RNA isolation and microarray design

The plants of the DH lines were grown under the following conditions: 16h light and temperature between 18 °C and 21 °C. After a week, germinated seedlings were transplanted and randomly distributed over three different blocks. Five weeks after transplanting, the third and fourth leaves of each replicate were collected in the morning (10:00–12:00h) and placed in liquid nitrogen to be further ground and stored at –70 °C. At that moment, none of the plants was switched to flowering/bolting, so all were still in the vegetative stage when sampling the leaves. Each replicate was ground individually and the mix with equally weighted amounts of the three replicates was used for RNA extraction and transcriptome profiling.

Total RNA was extracted by using the TRIZOL reagent (Invitrogen), from ~300mg of frozen leaf material of 92 DH68 lines. The first strand of cDNA was synthesized from 1 μg of total RNA using the I Amplification Grade kit (Invitrogen) according to the manufacturer’s instructions.

Agilent Cogenics 105K *Brassica* oligo-arrays (Agilent Technologies; Trick *et al.*, 2009), which contain 96 557 features, assembled using expressed sequence tag (EST) sequences mainly from *B. napus*, *B. rapa*, and *B. oleracea* resources, were used for two-colour microarray experiments. Distant pair design was applied for the two-colour microarray hybridization studies of DH lines as described by Fu and Jansen (2006) and implemented in the R package designGG (http://www.rug.nl/research/bioinformatics/). All microarray experiments were performed according to the manufacturer’s manual (Agilent Technologies).

### eQTL analyses

eQTL analysis was performed using the basic single marker regression procedure present in R/QTL. Expression was measured using two-colour array technology and, for the mapping, the ratios between two genotypes, Y_i_ = á+ âG_i_+Error (Y_i_=probe intensity, G_i_=genetic effect), were used. In this model, the genetic effect was annotated for the expression ratios as described in Fu and Jansen (2006); â is the effect of the different allele (1 for A>B 0 for A=B and –1 for A<B). This model was evaluated at each marker to get an estimate of the allelic effect on the expression probes. This resulted in a *P*-value, which was transformed into –log10 (*P*-value) (equivalent to LOD score). The eQTLs with LOD >3.0 were considered significant eQTLs. Several genetically linked markers with LOD >3.0 probably represent a single locus explaining variation in expression of the respective probe, but using this analysis these are described as several eQTLs. All 96 557 expression probes were annotated against the genome sequences of *B. rapa* (Wang *et al.*, 2011), by blasting their 60-mer oligonucleotide sequence against both genomic (scaffolds) and coding (gene models) sequences of the *B. rapa* Chiifu genome with the allowance of only one mismatch (http://Brassicadb.org/Brad/search All.php). eQTLs (local eQTLs) were defined as *cis* when the eQTL mapped on the same chromosome as the physical location of the oligonucleotide, and as *trans* eQTLs (distant eQTLs) when the eQTL mapped on a different chromosome from the physical location of the probe.

### Quantitative real-time PCR (RT-qPCR)

Expression of 16 genes was analysed using real-time PCR. A number of genes were selected because they were important Ft genes that were not represented on the Cogenics Microarray. These genes were: *FT*_A02, *FT*_A07(1), *FT*_A07(2), *SOC1*_A03, *SOC1*_A04, *SOC1*_A05, *CO*_A10, and *CCA1*_A05, and the two *FLC* paralogues *FLC2*_A02 and *FLC3*_A03. Real-time PCR was performed with paralogue-specific primers for all four *BrFLC* genes, and this was compared with microarray expression values for *FLC1*_A10 and *FLC5*_A03. A few other single-copy genes with eQTLs detected using the microarray profiles were selected to compare results obtained from the microarray and real-time PCR (*ARR3*_A09, *FRL2*_A09, and *CAM1*_A07). In addition, two single candidate Ft genes *BrCCA1* and *BrLD*, which had no eQTLs on the microarray, were also examined by RT-qPCR. Transcripts of all 16 genes were profiled with two technical replications using the RNA samples of the 92 DH68 lines that were previously used for microarray analysis. RT-qPCR was carried out in 96-well optical reaction plates using the iQ™ SYBR^®^ Green Supermix (Bio-Rad, www.Bio-rad.com). All the cycle threshold (*C*_*t*_) values from one gene were determined at the same threshold fluorescence value of 0.2 using the ΔΔ*C*_*t*_ method (Livak and Schmittgen, 2001). The reference gene glyceraldehyde-3-phosphate dehydrogenase (*GAPDH*) and gene-specific primers for the RT-qPCR are listed in Supplementary Table S3 at *JXB* online.

### Expression correlation and genetic network analyses

Pearson’s correlation among Ft genes with eQTLs was computed based on microarray signal intensities (log_2_ scale), RT-qPCR (log_2_ values), and flowering time phenotypes (days) of each DH line to identify co-expression patterns among gene probes and flowering time. Using the heat map tool, including hierarchical clustering among the genes and flowering time phenotype, the correlation between gene expression and phenotype was visualized.

Similarly, a gene network based on co-regulation patterns among genes was constructed to identify regulatory gene hubs of flowering time pathways. Pearson’s correlation was calculated by using eQTL LOD profiles of genes represented in the microarray, RT-qPCR values, and flowering time phenotype; correlations >0.3 between genes were shown in the network. Visualization of the co-regulation network was carried out by using the open source visualization software Cytoscape 2.8.2 (www.cytoscape.org) to analyse the genetic interaction networks (Shannon *et al.*, 2003). The calculations of Pearson’s correlation and heat map was done in open statistical software R 2.13.1 (Ihaka and Gentleman, 1996).

## Results

### Mapping of flowering time genes

**Fig. 1.**
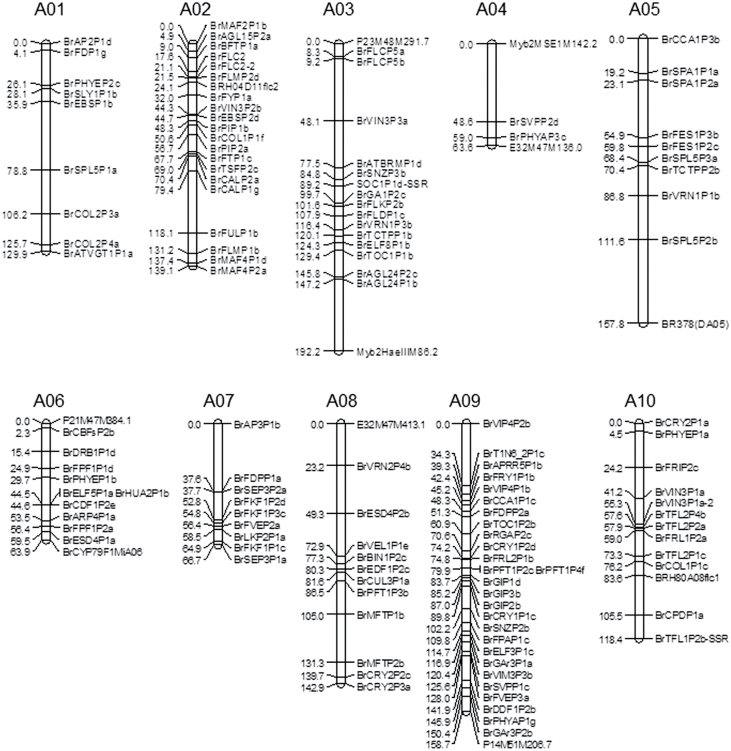
The genetic map (cM) of DH68 enriched with flowering time candidate genes. The flowering time candidate gene markers are shown <Genus species, Br> <Gene name, 2–6 letter code> <Genome locus, Paralogues code> <Primer code, letter code>. The end marker of link groups is indicated by another genetic marker if the end marker is not a flowering time marker.

A total of 768 *B. rapa* genes were identified in a screen of EST, bacterial artificial chromosome (BAC), and Chinese cabbage genome sequences as homologues of 365 genes involved in flowering in *Arabidopsis* (Table 1; Supplementary Table S1 at *JXB* online). Of the *Arabidopsis* genes, 101 (27.7%) had three or more paralogues in *B. rapa*, 128 (35.1%) had two *B. rapa* paralogues, 136 (37.3%) had one *B. rapa* homologue, and for 11 (3.0%) *Arabidopsis* genes no homologues were identified in *B. rapa*. A total of 190 gene/paralogue-specific primer pairs designed to amplify *B. rapa* genes amplified polymorphic bands between the parents of the DH68 population (YS-143 and PC-175). These 190 polymorphic primer pairs were profiled over the DH68 mapping population, which resulted in map positions for 125 homologues of Ft genes in *B. rapa*, corresponding to 81 Ft genes from *Arabidopsis* (Fig. 1; Supplementary Table S2 at *JXB* online). These Ft genes were distributed over all 10 linkage groups, covering a total map length of 1233 cM. An updated genetic linkage map of DH68 contains 456 markers: 278 AFLPs, 50 SSRs, 125 Ft markers, 2 IBP (intron-based polymorphism), and 1 CAPS (cleaved amplified polymorphic sequence) (Supplementary Fig. S1 at *JXB* online).

**Table 1.** *The number of* Arabidopsis *orthologues encoding genes involved in flowering with* ≥*3, 2, 1, or 0 paralogues in* B. rapaIn *total, 366* Arabidopsis *orthologues detected 768* B. rapa *paralogues*

No. of *Arabidopsis* orthologues	No. of paralogues *in B. rapa*	Percentage
101	≥3	27.6
129	2	35.3
136	1	37.3
11	0	3.0

All 768 *B. rapa* genes homologous to *Arabidopsis* Ft genes were *in silico* mapped on scaffolds and chromosomes of the sequenced Chinese cabbage Chiifu genome. For 40 of the 768 Ft genes, no *in silico* map position could be found. The genetic map position of Ft genes in DH68 corresponded well with their *in silico* predicted map position in Chiifu. Only 12 genes had an inconsistent order between the genetic and physical maps, but these were still mapped to the same linkage groups (Supplementary Fig. S2 at *JXB* online). The relationship between the physical and genetic distance in this euchromatic sequenced part of the genome was 1 cM= ~210kb.

### Flowering time QTL analysis

**Fig. 2.**
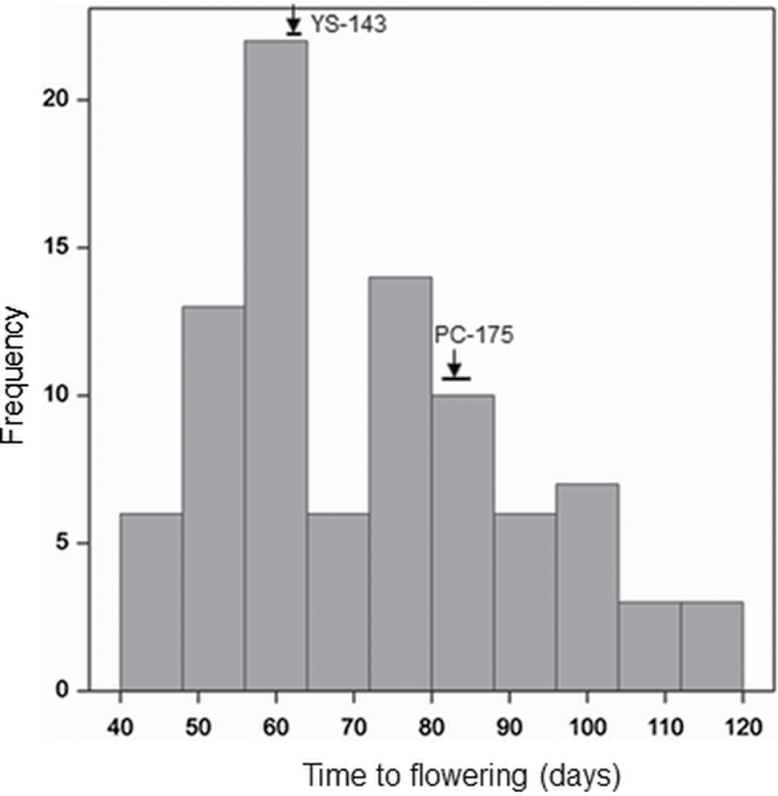
Frequency distributions of flowering time in DH68. Arrows and horizontal bars depict the mean ±SD of parental lines.

**Fig. 3.**
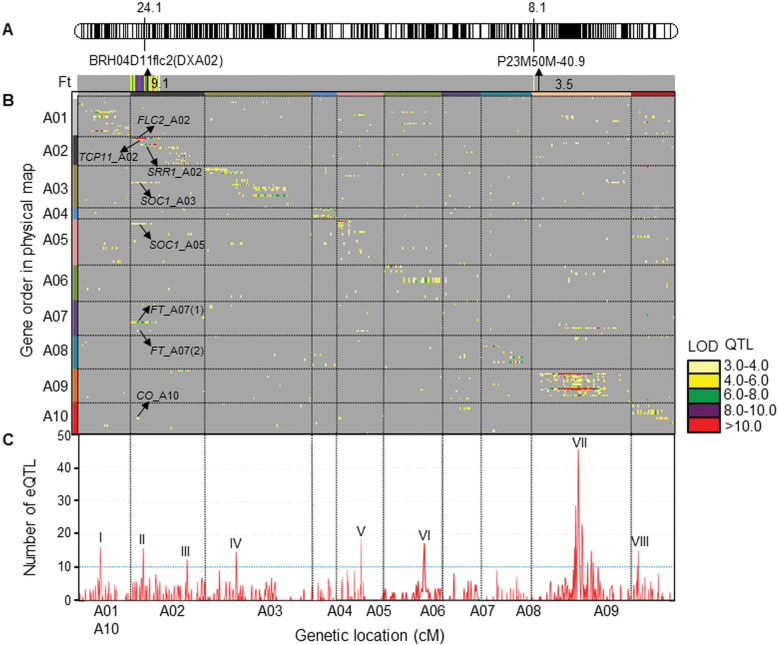
Summary of flowering QTLs, eQTLs, and hotspots of eQTL locations detected on the genome in *B. rapa* for flowering time. (A) Location of QTLs for flowering time in DH68 (YS-143×PC-175). The bar at the top represents the genetic map of the 10 linkage groups. The QTLs identified are shown as 1–LOD support intervals, the position of arrows corresponding to the maximum LOD score values. Map positions of flowering QTLs are given in centiMorgans (cM) above the genetic map, and the markers are listed below the genetic map. Upward arrows indicate the LOD score of the Ft QTLs on A02 and A09. (B) Genomic architecture of flowering time eQTLs across the chromosome of *B. rapa*. Chromosome borders are depicted as horizontal and vertical lines. The *x*-axis (cM) shows the genetic locations of markers mapped in the DH68 linkage map. The *y*-axis shows the location/order of probes representing candidate genes for which eQTLs were found. The displayed data are included as Supplementary Table S6 at *JXB* online. (C) Numbers of eQTLs in intervals of a 5 cM sliding window are given across the genome. The left *y*-axis shows the number of eQTLs, with the horizontal blue line showing the flowering time eQTL hotspot threshold of 10. The cut-off number of eQTLs by chance would be 10. Eight regions had a high eQTL density centring around ±0.5 cM. The eight eQTL hotspots were noted as I–VIII (arranged left to right).

The flowering time of DH68 lines showed a distribution around the mean, which deviated from continuous as the class around 70 d had fewer DH lines (Fig. 2). QTL mapping identified two loci affecting flowering time that together explained 42.6% of the phenotypic variation (Fig. 3A). A large percentage of phenotypic variation (33.5%) was explained by a QTL on the top of A02 (24.1 cM) with a LOD value of 9.1. This QTL maps at the Ft gene *BrFLC2* locus. The other flowering QTL was located on A09 with a LOD value of 3.4 (8.1 cM), accounting for 9.1% of the phenotypic variation. For both QTLs, the Yellow sarson allele decreased flowering time (Fig. 3A).

### eQTL analysis based on the microarray study

In order to identify genes that underlie the flowering QTLs and to construct co-expression networks that relate to flowering time, a regression analysis of the transcript abundance in the 92 DH lines was performed for the 96 557 probes that were represented on the microarray against the 456 genetic markers. In total, 28 403 probes (29.4%) were detected as significant against at least 1–36 markers with a LOD >3, corresponding to a total of 160 803 eQTLs.

From the 96 557 probes on the microarray, 1340 probes represented 610 Ft genes in *B. rapa* (Supplementary Tables S4, S5 at *JXB* online). After regression analysis, 188 out of the 1340 probes (14.0%), corresponding to 178 Ft genes in *B. rapa*, had one or more significant eQTLs, and within these 114 (60.6%) had a *B. napus* origin, 63 (33.5%) had a *B. rapa* origin, 10 (5.3%) had a *B. oleracea* origin, and only one (0.5%) had a *B. carinata* origin (Supplementary Table S6 at *JXB* online). When the genetic positions of the markers were plotted against the physical positions of the features for which eQTLs were found, an almost linear genome-wide relationship along the diagonal of the graph was observed (Fig. 3B; Supplementary Table S6 at *JXB* online).

### eQTL analysis based on the RT-qPCR study

The expression of 17 important candidate genes for flowering time was measured by RT-qPCR, chosen as they were absent on the microarray and to verify the microarray results. Using the normalized relative abundance of transcripts, a total of 18 eQTLs for the expression level of 13 genes were mapped with significant LOD scores of at least 3 (Supplementary Fig. S3 at *JXB* online). *Cis*-acting eQTLs for three single-copy genes (*ARR3_*A09, *FRL2_*A09, and *CAM1_*A07) identified using the microarray were further confirmed by RT-qPCR, with higher LOD scores for RT-qPCR data (Supplementary Table S6 at *JXB* online). *BrFLC1* had a *cis* eQTL on A10, while a minor *trans* eQTL was only detected on A09 with real-time PCR. A summary of the peaks/positions and nearest markers of eQTLs with LOD scores and explained variance is displayed in Table 2.

**Table 2.** Details of eQTLs detected based on the relative transcript abundance of 11 genes obtained by RT-qPCR

Gene	Peak of eQTL on LG (cM)	Interval (cM)^*a*^	Nearest marker	LOD score	Variance explained (%)	Regulation	Array-eQTLs^*b*^	Flowering QTLs
*FLC1*_A10.RL	(A09) 128.2	123.4–129.2	P14m51m321.6	4.3	18.6	*trans*	ND	–
	(A10) 83.6	78.2–94.2	BRH80A08flc1 (DA10)	3.1	12.8	*cis*	A10	–
*FLC2*_A02.RL	(A02) 24.1	22.5–27.2	BRH04D11flc2 (DXA02)	24.6	71.4	*cis*	ND	A02
*FLC3*_A03.RL	(A03) 29.2	23.4–34.5	E37M47M128.1	4.9	22.7	*cis*	A03	–
*FLC5*_A03.RL	(A05) 54.9	51.1–58.9	BrFES1P3b (XSA05)	3.7	17.9	*trans*	A03	–
*FT*_A07(1).RL	(A02) 21.1	17.6–23.3	BrFLC2-2 (XSA02)	6.5	24.0	*trans*	ND	–
*FT*_A07(2).RL	(A02) 21.1	17.6–23.3	BrFLC2-2 (XSA02)	3.4	16.5	*trans*	ND	–
	(A05) 81.9	80.5–86.0	P14M51M121.9	3.0	12.3	*trans*	ND	–
*SOC1*_A03.RL	(A02) 48.3	46.7–49.9	BrPIP1b(XSA02)	4.7	17.9	*trans*	ND	–
	(A09) 128.2	116.9–129.2	P14M51M321.6	3.6	13.5	*trans*	ND	–
*SOC1*_A05.RL	(A02) 36.9	27.2–42.9	ENA13I (DA02)	4.1	16.4	*trans*	ND	-
*CO*_A10.RL	(A02) 21.5	16.3–29.8	BrFLMP2d (XSA02)	3.4	10.7	*trans*	ND	A02
	(A05) 19.2	5.0–28.1	BrSPA1P1a (XXA05)	4.2	13.6	*trans*	ND	–
	(A10) 76.2	64.2–82.2	BrCOL1P1c (XSA10)	4.9	16.1	*cis*	ND	–
*CCA1*_A05.RL	(A05) 23.1	22.2–32.1	BrSPA1P2a (XSA05)	22.1	69.0	*cis*	ND	–
*ARR3*_A09.RL	(A09) 66.5	65.6–68.9	E32M47M56.0	16.7	58.7	*cis*	A09	–
*FRL2*_A09.RL	(A09) 74.3	74.0–74.8	BrCRY1P2d (XXA09)	30.4	80.0	*cis*	A09	–
*CAM1*_A07.RL	(A07) 0.0	0.0–5.0	BrAP3P1b (XSA07)	3.0	14.6	*cis*	A07	–

^*a*^1.0 LOD confidence interval.

^*b*^ ND indicates that the gene was not represented on the Cogenics Microarray.

### Genome distribution of eQTLs

When expression profiles of eQTLs of the microarray (188 Ft probes/genes with eQTLs) and RT-qPCR (13 Ft probes/genes with eQTLs) were combined, a total of 201 Ft genes/probes revealed a total of 1145 significant marker–probe associations (919 from the microarray and 226 from RT-qPCR) against all 456 genetic markers (Table 3). Based on their genetic map position or physical map position relative to markers with genetic map positions, 58 genes/probes had *cis* eQTLs on the chromosome where they physically mapped, 86 genes/probes were *trans* regulated by loci on different chromosomes, while 40 genes/probes were both *cis* and *trans* regulated, and for 17 genes/probes it was unknown whether they were *cis* or *trans* regulated (Supplementary Table S6 at *JXB* online). To visualize eQTL hotspots, the density of eQTLs was calculated in a 5 cM sliding window (Fig. 3C). Eight eQTL hotspots I–VIII are distributed over seven linkage groups with 11–40 eQTLs/hotspots, regulating the transcription of Ft genes. Two of the eight hotspots were on A02, with 14 (24.1 cM on A02) and 11 (110.1 cM on A02) eQTLs. The largest hotspot (VII) had 40 eQTLs at 74.2 cM on A09. All these hotspots may contain key regulators: genes controlling the expression of many other genes.

**Table 3.** Number and proportions of significant eQTLs (marker–probe associations) with different LOD scores in different linkage groups

LOD level	3–4	4–6	6–8	8–10	>10	Total	Percentage
A01	50	38	8	4	2	102	8.9
A02	81	44	14	8	10	157	13.7
A03	87	59	11	2	0	159	13.9
A04	20	14	4	1	0	39	3.4
A05	47	20	1	1	4	73	6.4
A06	50	26	8	1	0	85	7.4
A07	25	14	0	0	0	39	3.4
A08	25	17	7	0	2	51	4.5
A09	163	100	24	17	52	356	31.1
A10	38	38	5	1	2	84	7.3
Total eQTLs	586	370	82	35	72	1145	

### Co-localization analysis of eQTLs and flowering QTLs

Subsequently, a co-localization analysis was performed between flowering QTLs and eQTLs (Fig. 3; Supplementary Fig. S2 at *JXB* online). A strong *cis* eQTL for *FLC2*_A02 on A02 at 24.1 cM (95% confidence interval) with a LOD of 25, explaining 71.4% of the *BrFLC2* transcript variability, co-localized with the major flowering QTL on A02 (Supplementary Fig. S2 at *JXB* online) with very similar shapes of the LOD plots (Supplementary Fig. S3 at *JXB* online). The YS-143 *BrFLC2* allele was associated with both decreased *BrFLC2* transcript abundance and decreased flowering time.

At this *FLC2*_A02 locus, several *trans* eQTLs were identified for *CO*_A10, *FT*_A07(1), *FT*_A07(2), *SOC1*_A03, and *SOC1*_A05, and *cis* eQTLs for *SRR1*_A02 and *TCP11*_A02 (Supplementary Fig. S3; Supplementary Table S6 at *JXB* online). As the confidence intervals co-localized with that of the *FLC2*_A02 *cis* eQTL, *BrFLC2* seems a major regulator for the expression of these genes and for flowering time in *B. rapa*.

At A09, a minor flowering QTL maps; no clear candidate Ft genes can be assigned as *cis* eQTLs map proximal to this flowering QTL. At chromosome A10, another *BrFLC* paralogue maps: *FLC1*_A10.RL with a *cis* eQTL. However, at this locus, no QTL for flowering time is identified (Supplementary Table S6 at *JXB* online).

### Identification of Ft gene modules

**Fig. 4.**
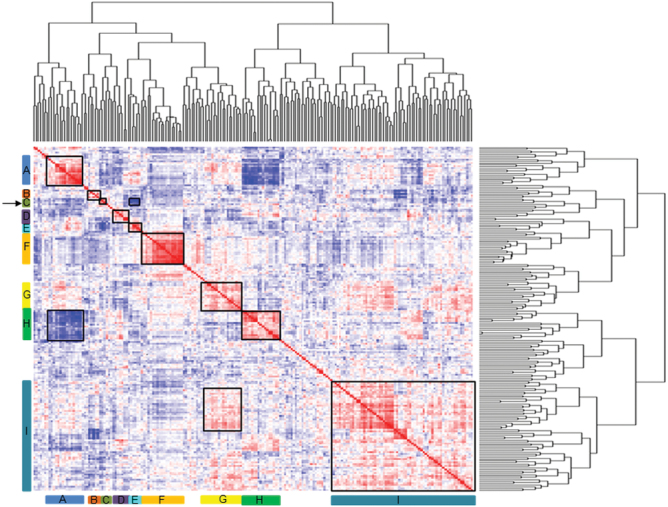
Correlation heat map of gene expression data using microarray and RT-qPCR, and flowering time phenotype (Ft, in days). Pearson’s correlation was calculated among 199 genes and the Ft phenotype to show the co-expression pattern of genes in the heat map. The agglomerative hierarchical cluster is shown alongside the heat map to illustrate grouping patterns and Ft phenotypes. Colour key indicates the correlation higher than absolute value 0.3 between genes: blue, negative correlation; red, positive correlation. The flowering time trait is located in block C, as indicated by the arrow. The displayed data are included as Supplementary Table S7 at *JXB* online.

To analyse the functional relevance of the gene expression signatures to the phenotypic traits, a correlation matrix was calculated based on expression values in log^2^ scale of 198 candidate Ft genes with eQTLs and the flowering time trait (days). The candidate Ft genes were classified into 11 functional pathways based on information available in the literature, and annotation: gibberellin (17), floral meristems (28), vernalization (30), autonomous (5), red/far red light signalling (16), photoperiod/circadian clock (51; these two classes were combined, consisting of nine circadian clock genes, 22 photoperiod genes, and 20 genes that were classified into both these pathways; Supplementary Fig. S4K at *JXB* online), regulation of transcription (2), light signalling (14), floral integrators (4), development (18), and unknown functional pathways (13). The heat map (Fig. 4; Supplementary Table 7 at *JXB* online) shows clear clusters of genes with correlated expression. For ease of description, these clusters were numbered from block A to block I, with I being a very large block in which smaller clusters with higher correlations can be distinguished. In block A, the transcript abundance of 15 genes mostly belonging the photoperiod/circadian clock functional pathway was significantly positively correlated among pairs of transcripts, and these were negatively correlated with block H, with 17 genes, nine from the photoperiod/circadian pathway and the remaining from five different functional pathways. Block B consists of only five genes that belong to as many different functional pathways, with negative correlation to block E genes. In the small block C, the vernalization pathway gene *FLC2*_A02 and the circadian clock gene *TCP11*_A02 were positively correlated with flowering time. Importantly, block C was significantly negatively correlated with block E that includes the vernalization pathway gene *FLC1*_A10, the floral development gene pistillata *PI*_A02, and the floral integrators *SOC1*_A05, *SOC1*_A03, *FT*_A07(1), and *FT*_A07(2). Block D is a small group of genes from several functional pathways. Block G contains genes from different functional pathways, with correlations between genes from other blocks, especially block I. Block H mainly consists of genes from the daylength/circadian pathways, with many negative correlations to genes from block A. The very large block I consists of 64 genes that belong to many different functional pathways. A cluster of genes can be identified with genes all mapping on A09, with, among others, correlations to the *BrCDF* and *BrGI* genes from the daylength/circadian pathways. As several blocks contain co-regulated genes that belong to different functional pathways, there is clear evidence for cross-talk between these pathways.

### Co-regulation network of Ft genes and flowering time

**Fig. 5.**
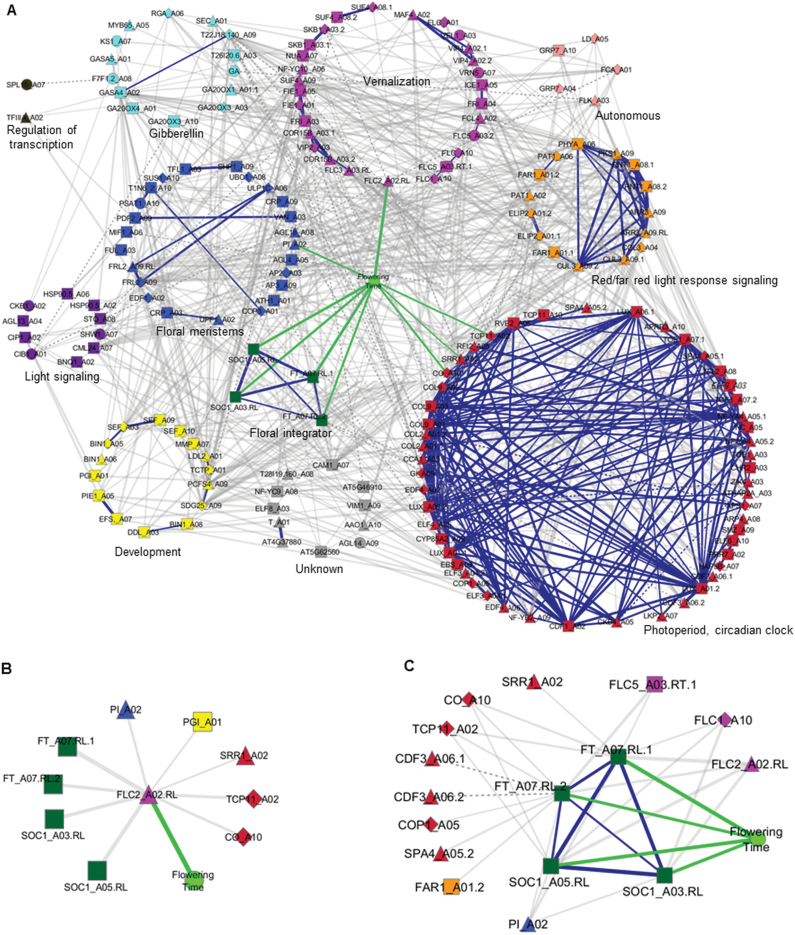
Co-regulation expression network of flowering time genes and flowering time in *Brassica rapa*. (A) Whole network visualization of correlation of all 197 flowering genes and flowering time phenotype. Genes were arranged in circles according to 11 different flowering time pathways. (B) A co-regulation subnetwork was extracted from the whole network focused on *FLC2*_A02.RL. (C) A co-regulation subnetwork was extracted from the whole network focused on the floral integrator pathway genes, which are all correlated to the flowering time trait. The correlation coefficients were calculated by using the LOD score values of 197 genes after eQTL mapping and of flowering time phenotype (Ft, in days) to visualize the co-regulation of flowering time pathway genes and phenotypic traits. The 197 Ft genes were from 11 functional pathways known for flowering time regulation. Only significant correlation coefficient values (*r* |>| 0.3) at a *P*-value of 0.05 were shown as edges in the network. The vertices of the network indicate genes or phenotype, and edges represent significant correlation. The grey coloured edges indicate the correlation coefficient between genes from different pathways, blue colour edges the correlation coefficient between genes within a pathway, and green colour edges the correlation coefficient between genes and flowering time phenotype. The wider the edge width, the higher the correlation coefficient. The solid lines indicate positive correlation and dotted lines indicate negative correlation. Shapes of the nodes indicate *cis/trans* regulation of genes (triangle, *cis*; square, *trans*; diamond, *cis*/*trans* regulation; and circle, unknown regulation), and a green coloured round-shaped node indicates the flowering time phenotypic trait. The displayed data are included as Supplementary Table S8 at *JXB* online.

To elucidate further how gene expression is regulated in leaves of DH68 lines and to identify genes/alleles underlying the flowering time QTLs, a co-regulation network was constructed. For this, a Pearson’s correlation matrix calculated based on LOD scores of 198 Ft genes with eQTLs and flowering time data of 2008 over all DH68 lines, was superimposed onto Cytoscape (a Pearson’s correlation was performed). The aim was to construct a co-regulation network in order to visualize correlations of the flowering time phenotype with Ft genes, grouped according to their functional pathways, and also to look at correlations among genes both within and between functional pathways and flowering time (Fig. 5A; Supplementary Table S8 at *JXB* online). *FLC2*_A02.RL had the highest correlation (*r*=0.89) with the flowering time phenotype. Of particular interest are four major floral integrator genes, namely *FT*_A07.RL.1, *FT*_A07.RL.2, *SOC1*_A03.RL, and *SOC1*_A05.RL, that are significantly correlated to each other, but also to the flowering time phenotype and *FLC2*_A02.RL. Similarly, the photoperiod/circadian clock genes (*CO*_A10, *TCP11*_A02, and *SRR1*_A02) and the floral meristem gene *PI*_A02 are significantly correlated with the flowering time phenotype (Fig. 5A).

In Fig. 5B, *FLC2*_A02.RL is depicted as a central node with strong correlation to the flowering time phenotype, and nine genes which also are intercorrelated; these genes are the floral integrators *SOC1*_A05.RL, *SOC1*_A03.RL, *FT*_A07.RL.1, and *FT*_A07.RL.2, the floral meristem identity gene *PI*_A02, and development and the photoperiod/circadian clock pathway genes *PGI*_A01, *SRR1*_A02, *TCP11*_A02, and *CO*_A10. These data clearly point to *BrFLC2* as a candidate gene for the major flowering QTL and key regulator of these nine Ft genes. The total network clearly shows that the genes in the photoperiod/circadian pathway are strongly correlated, while expression of genes in the red/far red response pathways also show strong intracorrelation. However, this is much less the case for all other functional pathways, for example genes in the vernalization pathway (Fig. 5A).

In order to better visualize interpathway correlations, every functional cluster inside the general gene network was extracted. This results in a presentation of the genes involved in these functional pathways with their correlations to genes from other pathways, resulting in a multitude of interactions and their connections to the flowering time phenotype (Fig. 5; Supplementary Fig. S4A–K at *JXB* online). For instance, based on the floral integrator functional pathway, a core subnetwork was revealed that connected to 12 nodes, containing three vernalization genes (*FLC1*_A10.RL, *FLC2*_A02.RL, and *FLC5*_A03. RL.1), seven photoperiod/circadian clock pathway genes (*SRR1*_A02, *CO*_A10, *TCP11*_A02, *CDF3*_A06.1, *CDF3*_A06.2, *COP1*_A05, and *SPA4*_A05.2), one red/far red light response signalling gene *FAR1*_A01.2, and one floral meristem gene *PI*_A02. All these genes were positively correlated, while the circadian clock genes *CDF3*_A06.1 and *CDF3*_A06.2 were significantly negatively correlated with *FT*_A07.RL.2 (Fig. 5C). Interestingly, looking at the genes in the unknown functional pathway, *CAM1*_A07 was clearly correlated to many genes from seven functional pathways and seems to be a major regulator (Supplementary Fig. S4J at *JXB* online). In *Arabidopsis*, *CAM1* encodes a calmodulin that is involved in thigmomorphogenesis (Chehab *et al.*, 2009). All of these subnetworks overlapped significantly with one another, illustrating the intercorrelation between many genes and pathways which all lead to the regulation of the flowering time phenotype in *B. rapa*.

### Differential expression of duplicated Ft genes

In order to understand the role of Ft genes that are duplicated in *B. rapa*, experiments were carried out to investigate whether paralogues of the same *Arabidopsis* genes had common *trans*-regulators. From the 201 Ft genes with eQTLs, 88 (44%) had ≥2 paralogues corresponding to 36 *Arabidopsis* orthologous Ft genes (Supplementary Table S9 at *JXB* online). From this set of genes, eight sets of paralogues had common *trans* eQTLs, while 16 had different *trans* eQTLs. The other 12 gene sets mapped *cis* to linked positions, and not to syntenic positions in the different subgenomes, and were not further analysed. The *BrFLC2* and *BrFLC3* genes were *cis* regulated, while *BrFLC1* was both *cis* and *trans* regulated, and *BrFLC5* was *trans* regulated.

## Discussion

The combined use of molecular markers, and phenotypic and gene expression data generated from a segregating population, offers the opportunity to detect phenotypic and expression QTLs from which co-expression networks can be generated that represent important components of flowering time regulation in *B. rapa*. In this study, a *B. rapa* genetic map with 456 markers was constructed (Supplementary Fig. S1 at *JXB* online), including 125 flowering time candidate gene markers, which are used as bridge markers between the genetic DH68 map and the Chinese cabbage physical map, which makes eQTL studies possible, as positional information allows determination of whether eQTLs are *cis* or *trans* regulated. The goal is to identify co-location between flowering time QTLs and eQTLs.

It is concluded that genes involved in flowering time regulation are preferentially maintained in *B. rapa*, as 27.7% of *Arabidopsis* Ft genes had ≥3 *B. rapa* paralogues/duplicated Ft genes, while for only 11 (3.0%) of all *Arabidopsis* genes were no homologues identified in *B. rapa*. In *B. rapa*, genome triplication followed by genome fractionation may have expanded gene families that underlie environmental adaptability, as observed in other polyploid species. Another explanation is the gene balance hypothesis, which states that genes encoding proteins that work in complexes are also less fractionated. As many Ft genes are transcription factors, that either work in complexes or are parts of the same modules, this gene balance hypothesis may also explain the high numbers of paralogues retained since polyploidization (Birchler and Veitia, 2010). In a recent publication, Lou *et al.* (2012) performed a detailed analysis of the circadian genes, a subset of the genes described herein, and concluded that circadian clock genes are preferentially retained relative to their neighbouring genes, a set of randomly chosen genes, and a set of housekeeping genes. They conclude that preferential retention of these circadian genes is consistent with the gene dosage hypothesis, which predicts preferential retention of highly networked or dose-sensitive genes.

When comparing the genetic order of the 125 Ft markers on the DH68 genetic map with the order of these markers based on the Chiifu genome sequence, there was in general a good fit, with few exceptions (Supplementary Fig. S2 at *JXB* online). At present, it cannot be concluded whether the inconsistency in order of a few markers between the genetic and physical map is because of incorrect map positions, or whether this is caused by genome rearrangements that differentiate the reference genome Chiifu and the two parental genotypes used in this cross.

Flowering time data are presented for the DH68 lines, progeny from a cross between Yellow sarson (YS-143) and Pak choi (PC-175). In total, two genomic regions with flowering QTLs were identified in this study, the two flowering QTLs were also reported in previous studies (Lou *et al.*, 2007; Li *et al.*, 2009; Zhao *et al.*, 2010). Co-location of flowering QTLs on A02 with the Ft gene *BrFLC2* is the first indication that allelic variation in this gene is the cause for the flowering QTLs. Additional evidence is provided by co-localization of the eQTL for *BrFLC2* with the major flowering QTL on A02 (Fig. 3).

In *Arabidopsis*, *FLC* acts as a dosage-dependent repressor of flowering, whose expression is down-regulated by vernalization (Michaels and Amasino, 1999). Evidence accumulated that the *FLC* homologues in *Brasscia* species act similarly to *AtFLC* and play a central role in the repression of flowering time (Schranz *et al.*, 2002; Lou *et al.*, 2007; Li *et al.*, 2009; Zhao *et al.*, 2010). Zhao *et al.* (2010) measured *BrFLC2* expression during plant development after varying periods of vernalization and demonstrated that seedling vernalization decreases *BrFLC2* expression in a dosage-dependent matter, and that this effect is most evident at the seedling stage, where flowering time is already established. The *BrFLC* paralogue *BrFLC1* was mapped on A10, with an *FLC1*_A10.RL *cis* eQTL (83.6 cM, LOD=2.8 by microarray and LOD=3.1 by RT-qPCR), but at this locus no QTL for flowering time is identified in this study. However, in the study of Lou *et al.* (2007), using the reciprocal cross, a flowering QTL at this locus was identified in August 2005 only (2.7 LOD value, 18% explained variation). Li *et al.* (2009) mapped QTLs for bolting, budding, lobe depth ratio, and flowering time that co-localized with the *BrFLC1* locus in an F_2_ mapping population from a cross between ‘Yellow sarson’ and ‘Osome’, a leafy vegetable. Allelic variation in *BrFLC1* also had an effect on flowering time when a Chinese cabbage collection was studied, caused by a splicing site variation in *BrFLC1* alleles (Yuan *et al.*, 2009). For the flowering QTL on A09, no clear candidate gene could be predicted among the genetic or *in silico* mapped *B. rapa* homologues of *A. thaliana* Ft genes. However, in this study, the possibility cannot be excluded that novel genes in *B. rapa*, possibly orthologues of *A. thaliana* genes without a role in regulating flowering time, play a role in regulating flowering time in *B. rapa*. It is possible that this A09 QTL can be explained by *B. rapa* genes that have no homology to *A. thaliana* flowering genes.

Figure 3 shows an overview of eQTLs and flowering QTLs. In Supplementary Fig. S2 at *JXB* online, the genetic and physical maps are aligned with the Ft genes without genetic map positions placed in intervals between mapped flanking markers; this facilitates the identification of *cis* eQTLs and genes underlying eQTLs and flowering QTLs. A clear diagonal is visualized, which demonstrates that the expression of many genes is *cis* regulated. As described in other studies (Schadt *et al.*, 2008; Holloway *et al.*, 2011; Jiang *et al.*, 2011), generally *cis* eQTLs exert stronger effects than *trans* eQTLs. In this study, *cis* eQTLs have LOD scores ranging from 3.0 to 30.4 (average 5.4, median 4.2), while *trans* eQTLs range from 3.0 to 58.6 (average 4.3, median 3.5). Forty-eight Ft genes (represented by 103 features) had eQTLs that mapped to A09; both *cis* and *trans* regulated (Supplementary Table S6 at *JXB* online). This implies that the expression of many (Ft candidate) genes (103 features out of 768 features, 13.4%) is genetically regulated by *trans*-acting regulators on A09. Remarkably, only one minor flowering QTL is detected on A09, proximal to the QTL hotspot (Table 3; Supplementary Fig. S3C at *JXB* online). It is also remarkable that from the eight flowering time eQTL hotspots, only the one on A02 co-localizes with a major flowering time QTL, while for the other loci it has to be concluded that regulatory hotspots for expression differences in Ft genes do not affect the flowering time itself in this experiment. It was investigated whether these flowering time eQTL hotspots co-localize with flowering time QTLs identified in other experiments using the same or other populations. This was not the case for DH68, but for its reciprocal cross DH38 (PC-175×YS-143) and for DH30 (Japanese vegetable turnip×common parent YS-143), flowering QTLs were detected on corresponding positions on A01, A03, A06, and A10; however, flowering QTLs have also been detected on A05 and A09 without eQTL hotspots (Lou *et al.*, 2007).

In several cases, paralogues of the same genes, such as *BrSOC1*, *BrFT*, but also *BrFLC*, are regulated by the same *trans*-acting factors (Supplementary Tables S7, S9 at *JXB* online). Overall, however, only 33% (eight) of the paralogue sets of genes had common *trans* eQTLs, while 67% (16) had different *trans* eQTLs in this study.

Flowering time in days, gene expression profiles of leaves from 5-week-old DH lines, and physical and genetic map positions of Ft genes were jointly analysed. This led to the detection of two QTLs for flowering time, with one major flowering QTL on A02 mapping at the *BrFLC2* locus, which points to a central role for *BrFLC2* in regulating flowering time, as there is a major *cis* eQTL for *BrFLC2*, *trans* eQTLs for floral integrator genes *BrFT* and *BrSOC1* and the daylength/circadian rhythm genes *BrCO*, and *cis* eQTLs for *BrSRR1* and *BrTCP11* (Fig. 5A).

The MADS domain transcription factor suppressor of overexpression of constans (*SOC1*) is a floral integrator. Several MADS domain proteins, including *SOC1* heterodimers, are able to bind *SOC1* regulatory sequences, while *SOCI* also binds to many flowering time regulatory and floral homeotic genes, which also bind to the *SOC1* regulatory sequences. Data of Immink *et al.* (2012) proved that *SOC1* constitutes a major hub in the regulatory networks underlying both floral timing and flower development. The *TCP11* protein is found to interact with different components of the core circadian clock, while *SRR1* plays an important role in phytochrome B light signalling and is also required for the normal clock function (Giraud *et al.*, 2010; Staiger *et al*., 2013). Mutations or allelic variation in the circadian clock genes can also alter flowering time (Fornara *et al.*, 2010). This is through the fact that circadian clock genes ensure that *CO* transcription peaks late in the day, which is enhanced by *GI* under long-day conditions. If *CO* transcription shifts to earlier in the day, *CO* is activated not only in long days but also in short days, which leads to earlier *FT* transcription and flowering under short-day conditions. *Arabidopsis SRR1* mutants had only subtle early flowering phenotypes in long days, but flowered much earlier in short days compared with the wild type. Experiments under different daylength regimes for DH68 would be interesting to compare gene co-expression networks.

The ultimate proof for the role of *BrFLC2* in flowering time regulation is expression of the PC-175 late allele in YS-143, and to assess flowering time phenotype. *Brassica rapa* is, however, extremely recalcitrant to transformation, and even though transgenic Chinese cabbage has been reported, no reports of transgenic oil types are published (Jung *et al.*, 2012; Park *et al.*, 2012). Allelic variation of the *BrFLC2* gene was determined by sequencing both parental (PC-175 and YS-143) alleles. In the YS-143 allele, a deletion of 56bp was detected at the exon 4 (12bp) and intron 4 (44bp) junctions (Supplementary Fig. S5 at *JXB* online). The PC-175 sequence did not have this deletion and was identical to the reference Chinese cabbage sequence in this region. Wu *et al.* (2012) sequenced *BrFLC2* alleles from Yellow sarson (accession L143), three Chinese cabbage genotypes, Rapid cycling (accession L144), Mizuna, Neep greens, Turnip, and Caixin, and identified a discontinuous 57bp insertion/deletion (InDel) across exon 4 and intron 4 in Yellow sarson resulting in a non-functional allele (Wu *et al.*, 2012). This deletion was absent in all other vegetable accessions they tested and present in many of the tested oil types, and homozygous in the three Yellow sarson accessions tested, but absent in the other accessions they tested. This deletion in *BrFLC2* may be the cause of the major flowering time QTL on A02, and may mask allelic differences of other *BrFLC* paralogues.

In conclusion, the data presented show that genes involved in flowering time are preferentially maintained in *B. rapa*, as the percentage of *Arabidopsis* Ft genes with ≥3 *B. rapa* paralogues was three times more (27.6%) than this percentage for all *Arabidopsis* genes (10.2%). The combined genetic analysis of flowering time and gene expression profiles in leaves from 5-week-old non-flowering DH lines from a cross between an oil type and a leafy type *B. rapa* led to identification of *BrFLC2* as a candidate gene for a major flowering QTL on A02, and as a *trans*-regulator of important floral integrator genes *BrSOC1* and *BrFT*, and key daylength/circadian rhythm genes *BrCO*, *BrSRR1*, and *BrTCP11*. This showed that orthologues of *A. thaliana* Ft genes play similar roles in *B. rapa*, and that for the genetic analysis of flowering time in *B. rapa*, *Arabidopsis* is a good model system.

## Supplementary data

Supplementary data are available at *JXB* online.

Figure S1. An updated genetic linkage map constructed with 125 candidate Ft gene target markers (GT) based on the DH68 population (YS-143×PC-175).

Figure S2. Ft genes as anchors between the genetic map (YS-143×PC-175) (cM) and physical map (Mb).

Figure S3. The relative abundance of 13 candidate Ft gene transcripts using RT-qPCR, plotted at 1 cM distances along all linkage groups.

Figure S4. Extraction of functional clusters from the general gene network.

Figure S5. *BrFLC2* nucleotide sequence (DNA) analyses within exon 4–exon 5 and comparison with *Arabidopsis thaliana* (cDNA) and the reference genome Chiifu *BrFLC2*.

Table S1. Selected flowering time genes.

Table S2. The sequence-informative markers of the flowering time genes mapped on a genetic linkage map.

Table S3. Primers for real-time RT-PCR.

Table S4. Annotation of the flowering time genes used in the microarray.

Table S5. Annotation of the sequence of flowering time genes in the microarray.

Table S6. Detailed information for eQTLs and pQTLs identified in this study.

Table S7. Detailed co-expression correlation values.

Table S8. The LOD correlation matrix of eQTLs.

Table S9. eQTL location of differentially duplicated paralogues/probes for the Ft gene set (2–8 copies).

Table S10. List of the physical order all Ft candidate genes and eQTLs mapped on their genetic locations.

Supplementary Data
