# Supplementary material for: The Brassica rapa FLC homologue FLC2 is a key regulator of flowering time, identified through transcriptional co-expression networks
Source: J Exp Bot. 2013 Sep 27;64(14):4503–16. doi: 10.1093/jxb/ert264 (PMC3808329; doi:10.1093/jxb/ert264)
Supplement: Supplementary Data [file supp_64_14_4503__index.html]

The Brassica rapa FLC homologue FLC2 is a key regulator of flowering time, identified through transcriptional co-expression networks — Supplementary Data 

# The *Brassica rapa FLC* homologue *FLC2* is a key regulator of flowering time, identified through transcriptional co-expression networks

## Supplementary Data

Data files

**Files in this Data Supplement:**

- Supplementary Data - Supplementary Data
- Supplementary Data - Supplementary Data
