# Supplementary material for: The Brassica rapa FLC homologue FLC2 is a key regulator of flowering time, identified through transcriptional co-expression networks
Source: J Exp Bot. 2013 Sep 27;64(14):4503–16. doi: 10.1093/jxb/ert264 (PMC3808329; doi:10.1093/jxb/ert264)
Supplement: Supplementary Data [file supp_ert264_Supplemental.pptx]

## Slide 1
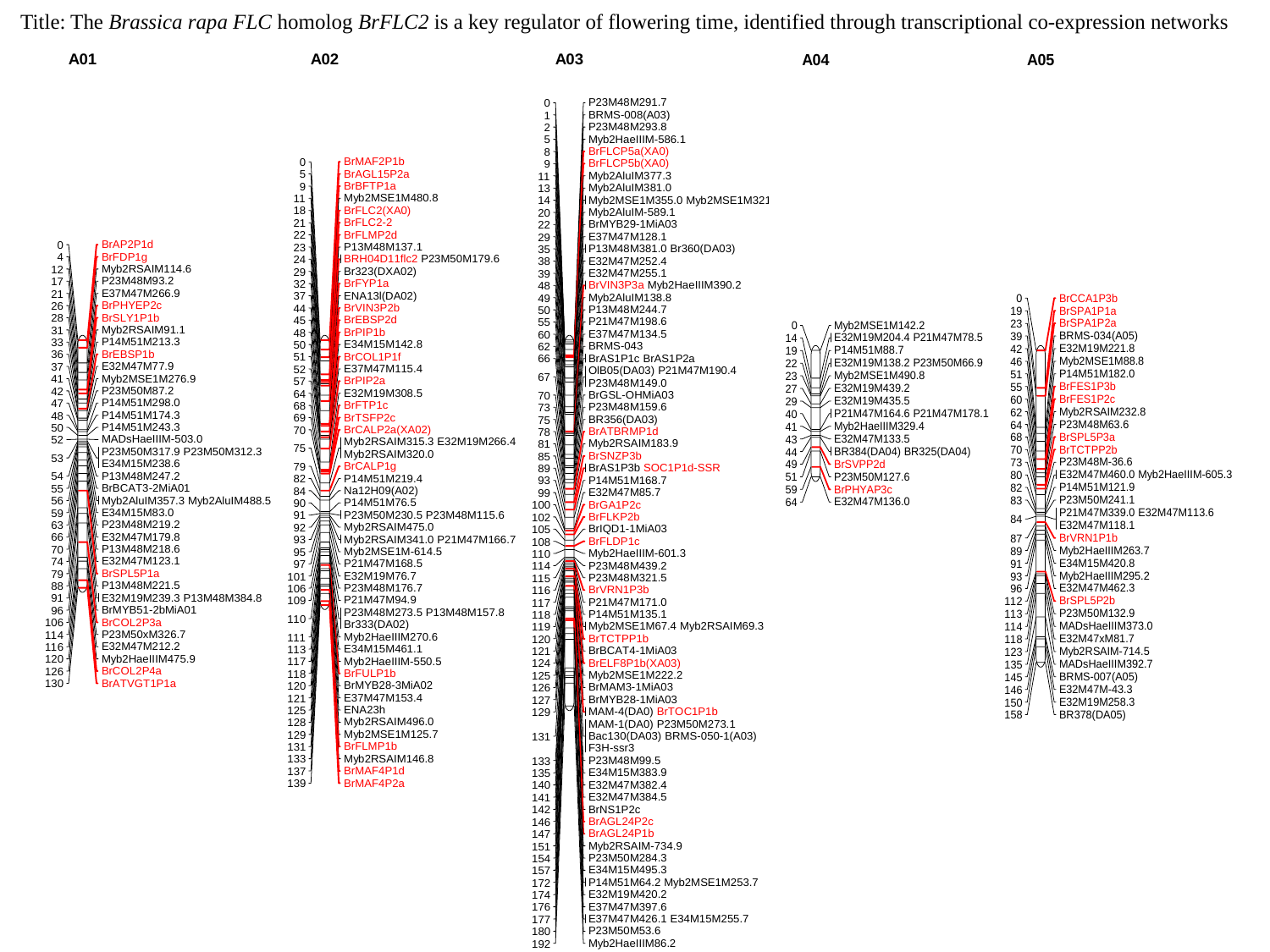

Title: The Brassica rapa FLC homolog BrFLC2 is a key regulator of flowering time, identified through transcriptional co-expression networks

## Slide 2
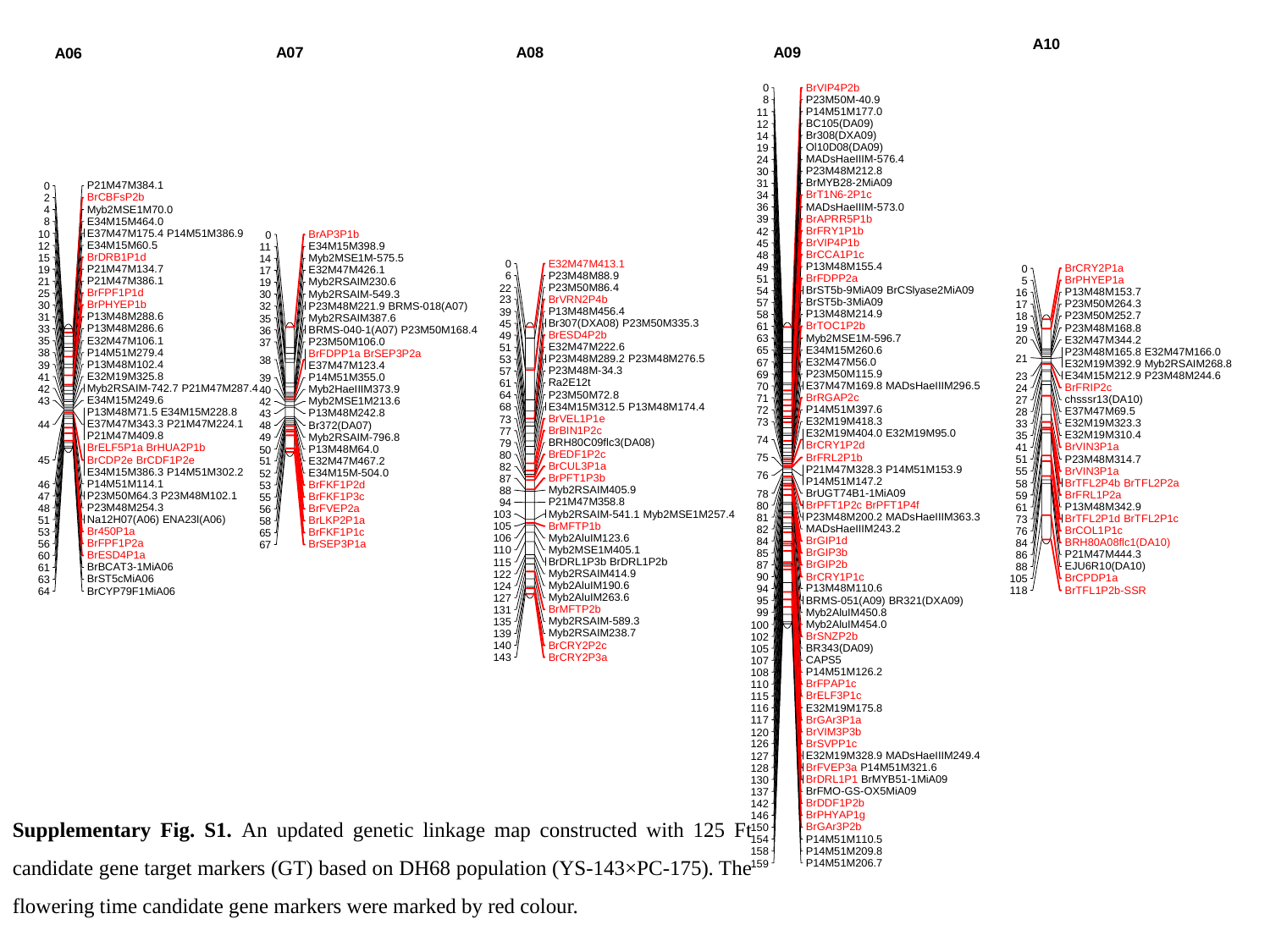

Supplementary Fig. S1. An updated genetic linkage map constructed with 125 Ft candidate gene target markers (GT) based on DH68 population (YS-143×PC-175). The flowering time candidate gene markers were marked by red colour.

## Slide 3
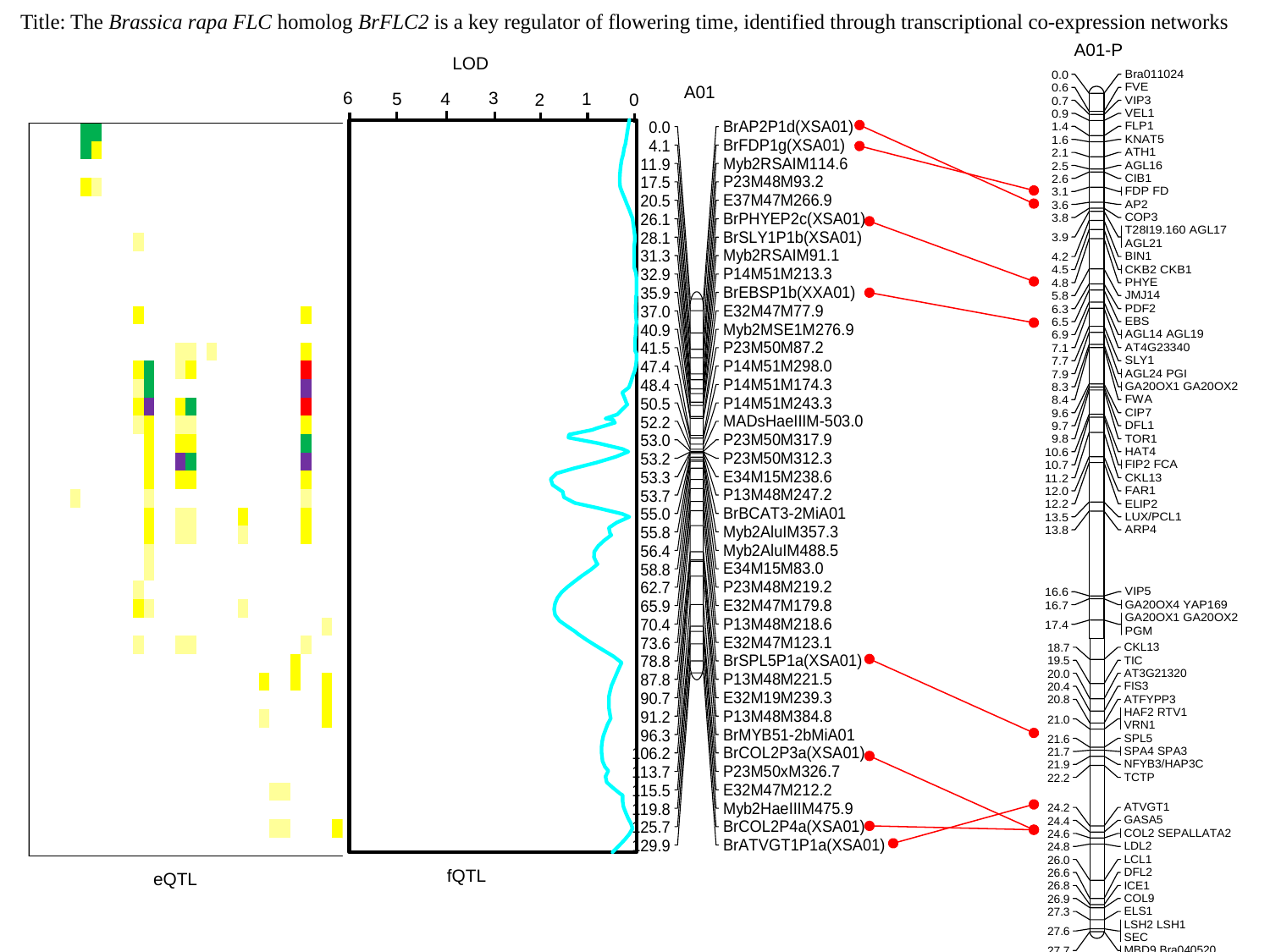

Title: The Brassica rapa FLC homolog BrFLC2 is a key regulator of flowering time, identified through transcriptional co-expression networks
A01-P
A01
3
6
4
1
2
0
fQTL
eQTL
LOD
5

## Slide 4
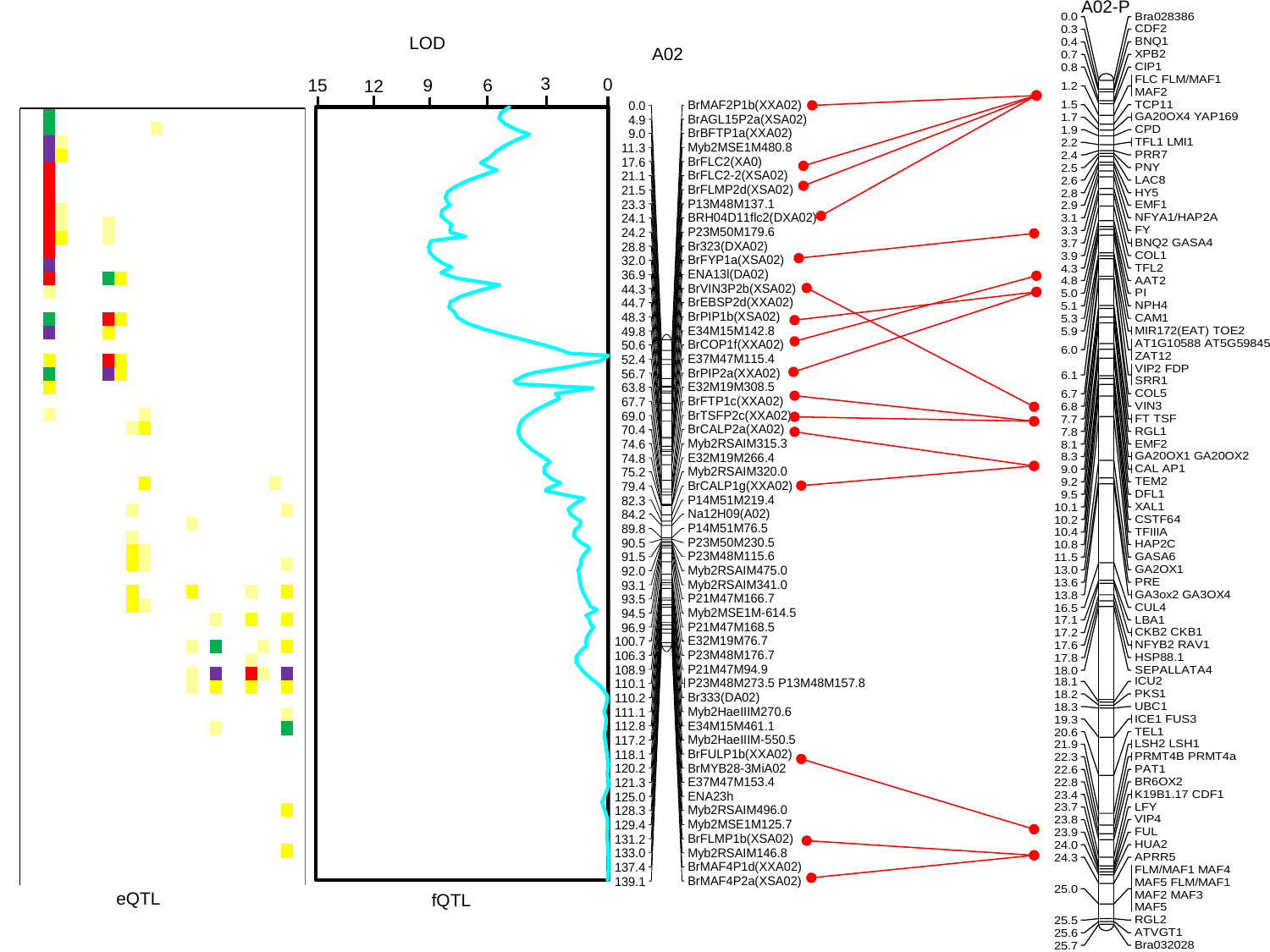

A02-P
A02
3
0
15
6
12
LOD
eQTL
fQTL
9

## Slide 5
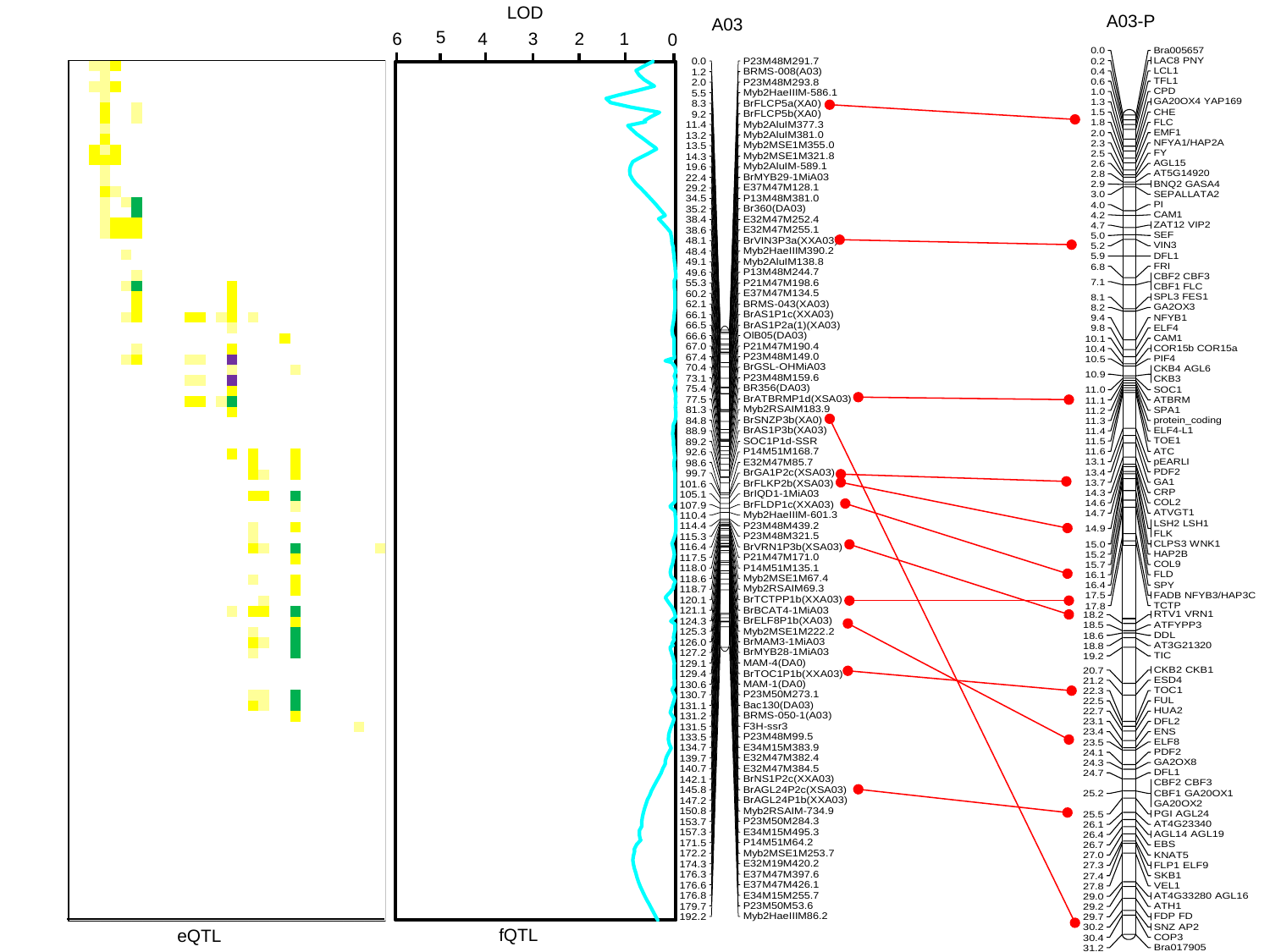

LOD
A03-P
A03
5
3
4
2
1
0
fQTL
eQTL
6

## Slide 6
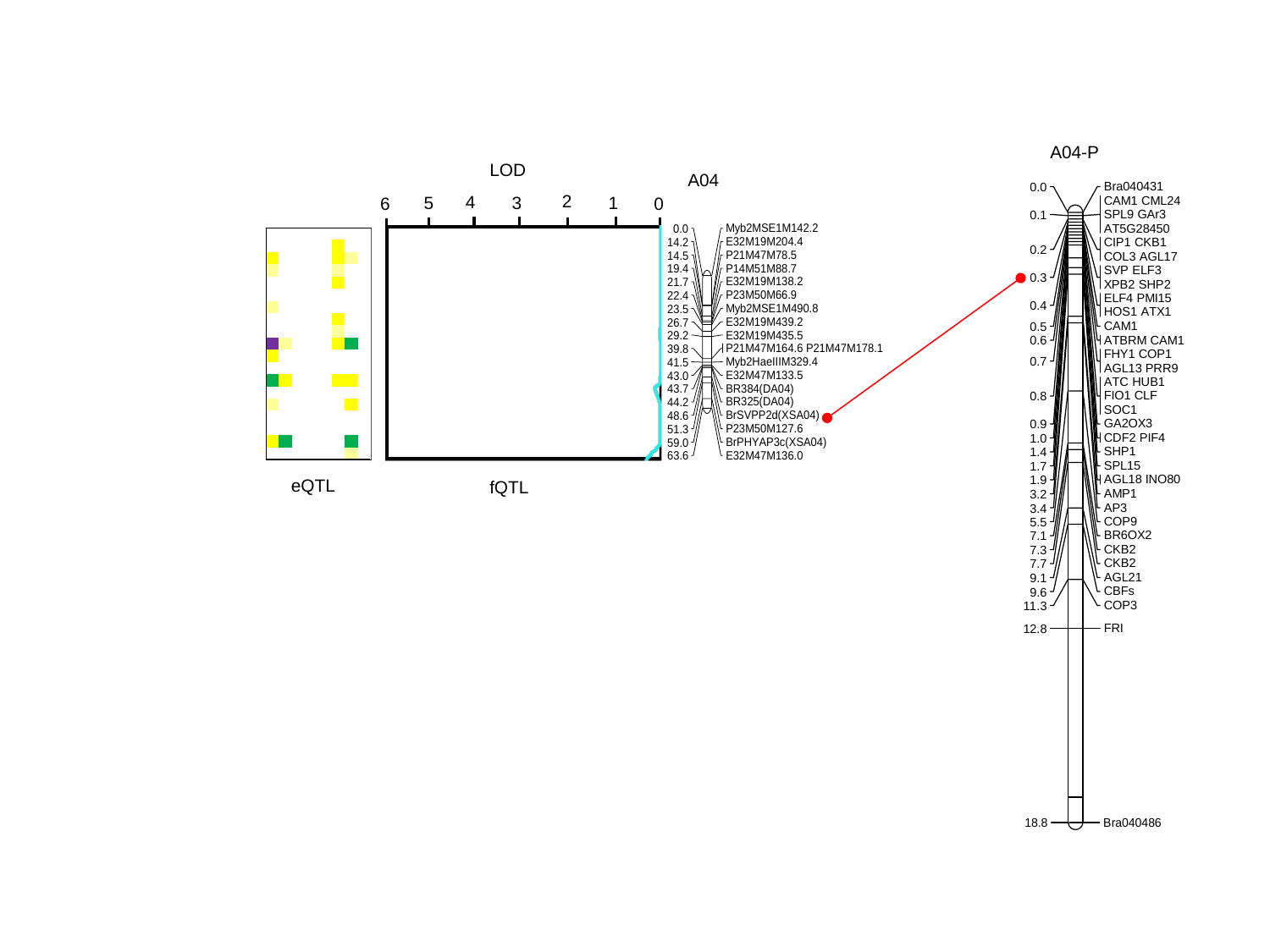

A04-P
A04
LOD
2
4
5
1
3
0
eQTL
fQTL
6

## Slide 7
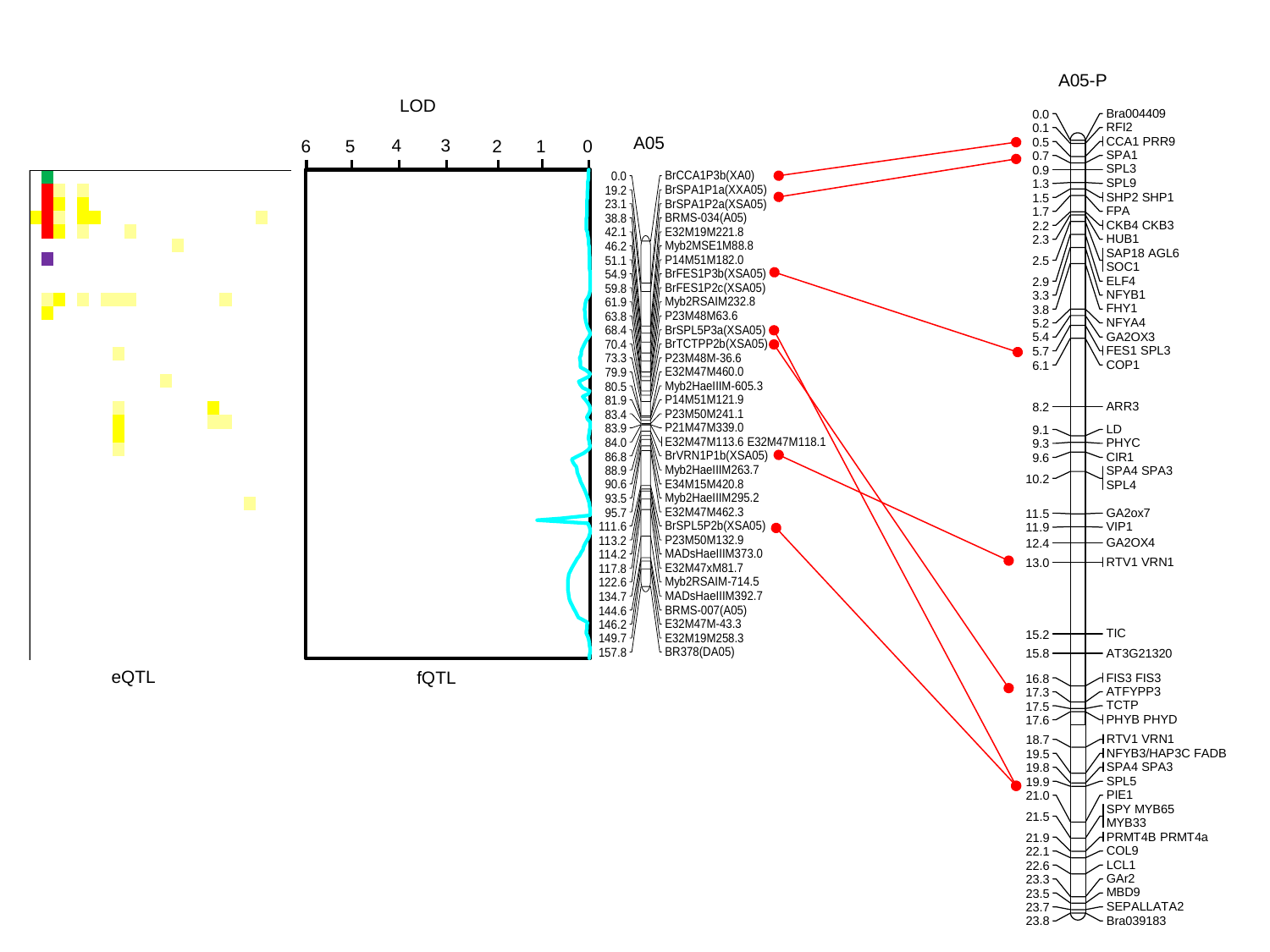

A05-P
A05
LOD
4
3
1
2
0
5
eQTL
fQTL
6

## Slide 8
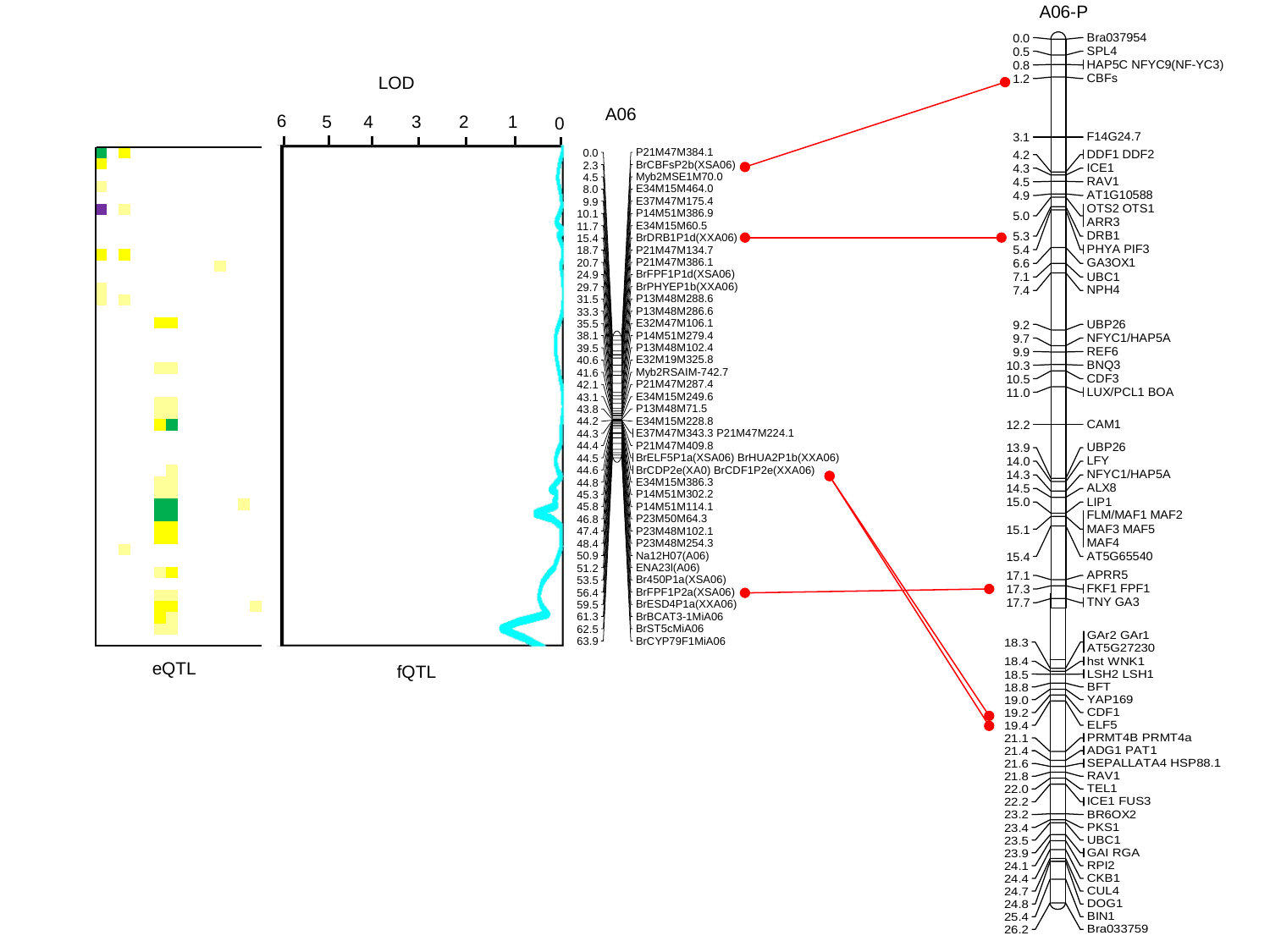

A06-P
A06
LOD
4
3
5
2
1
0
eQTL
fQTL
6

## Slide 9
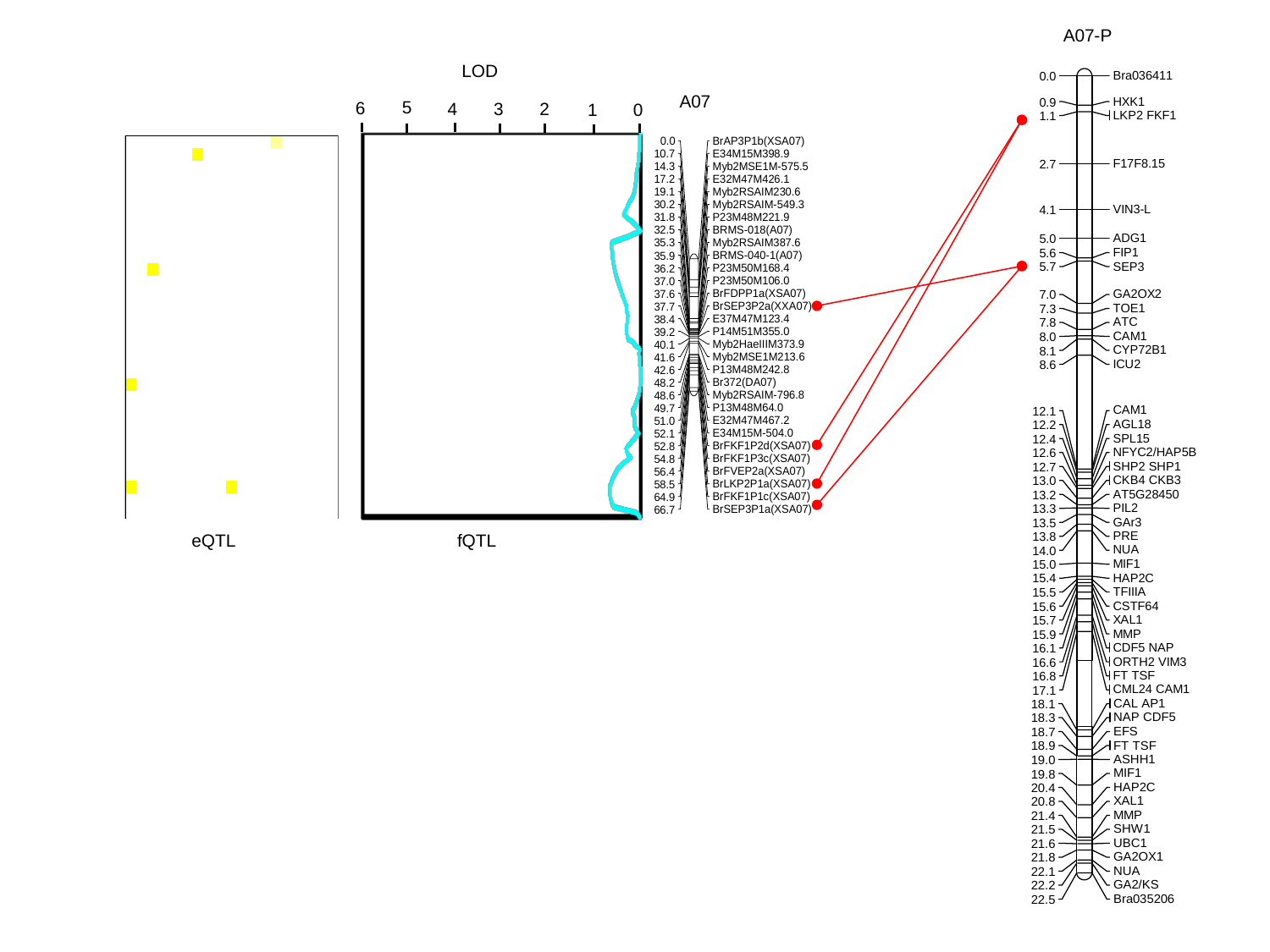

A07-P
A07
LOD
5
2
3
4
0
1
eQTL
fQTL
6

## Slide 10
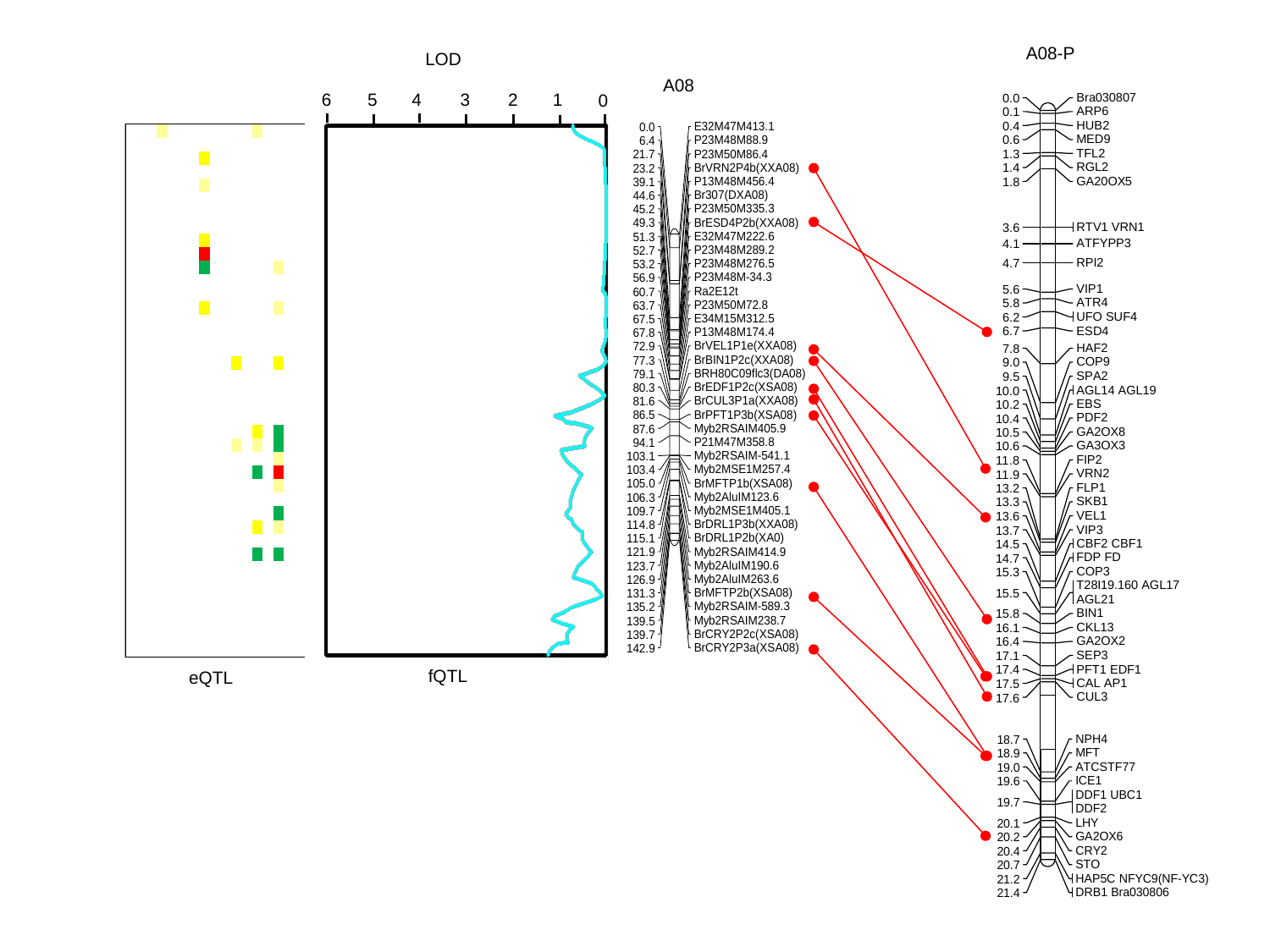

A08-P
A08
LOD
5
2
1
4
3
0
fQTL
eQTL
6

## Slide 11
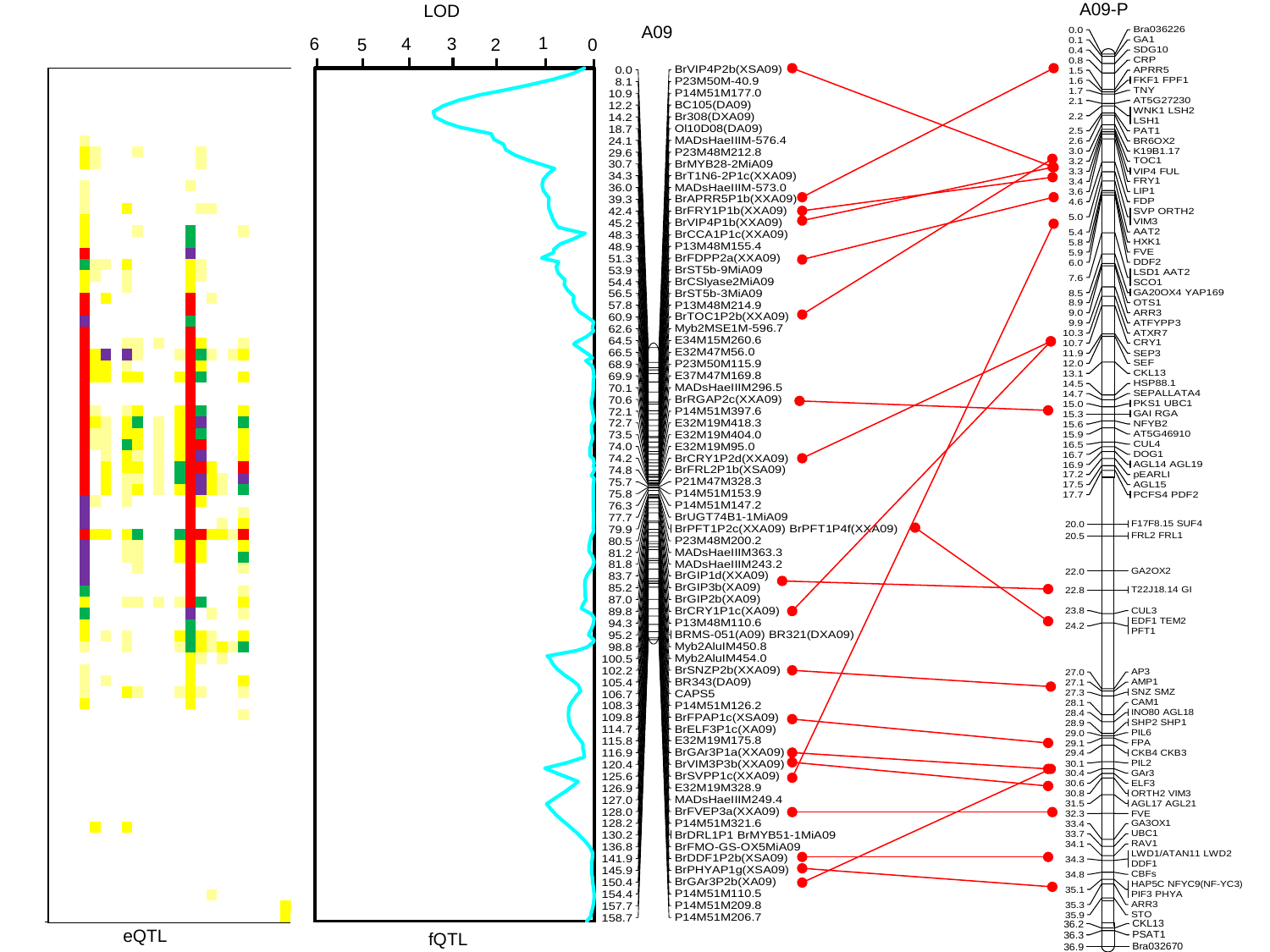

A09-P
A09
LOD
1
4
3
5
2
0
eQTL
fQTL
6

## Slide 12
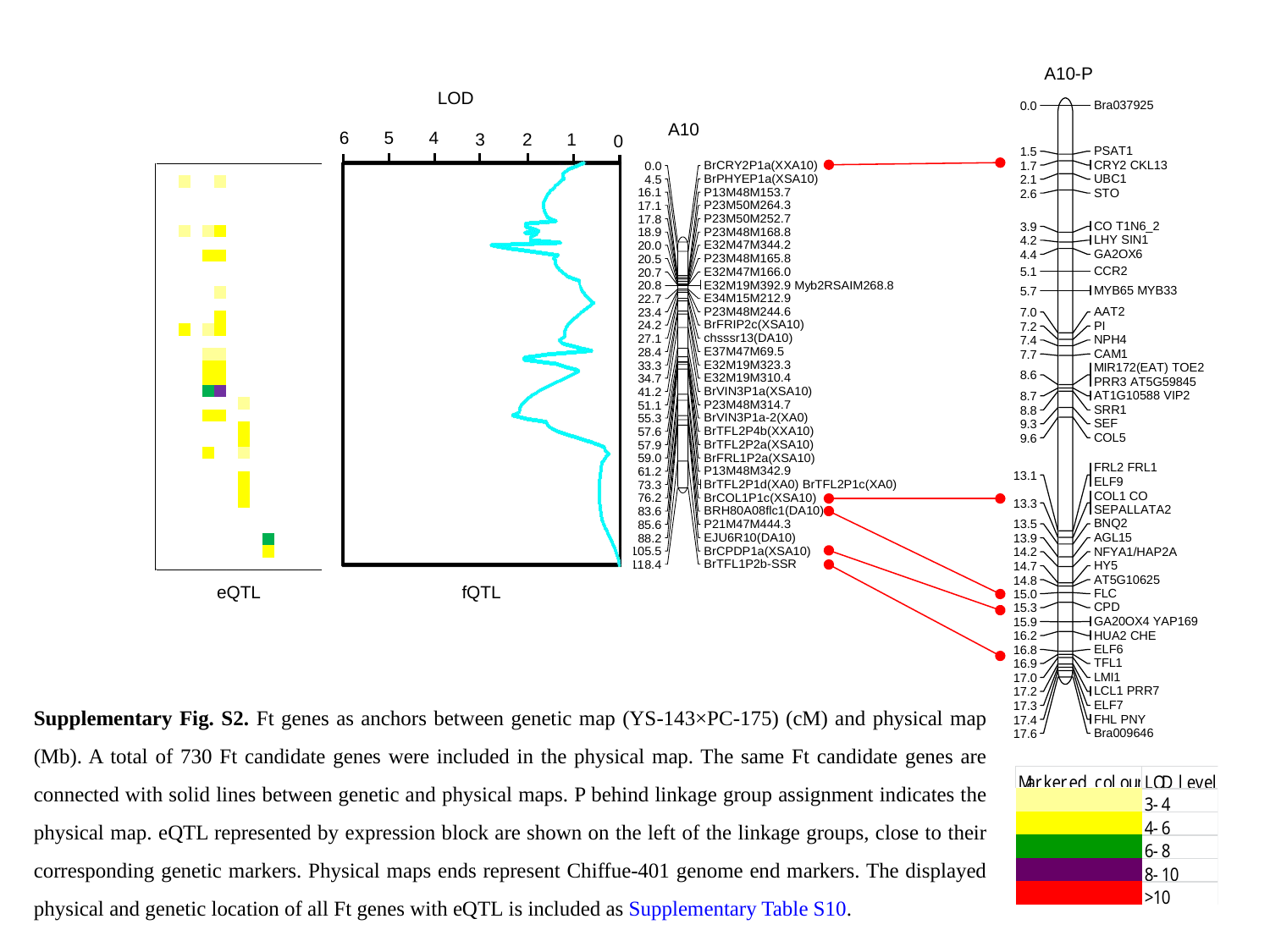

A10-P
A10
LOD
5
4
2
1
3
0
eQTL
fQTL
6
Supplementary Fig. S2. Ft genes as anchors between genetic map (YS-143×PC-175) (cM) and physical map (Mb). A total of 730 Ft candidate genes were included in the physical map. The same Ft candidate genes are connected with solid lines between genetic and physical maps. P behind linkage group assignment indicates the physical map. eQTL represented by expression block are shown on the left of the linkage groups, close to their corresponding genetic markers. Physical maps ends represent Chiffue-401 genome end markers. The displayed physical and genetic location of all Ft genes with eQTL is included as Supplementary Table S10.

## Slide 13
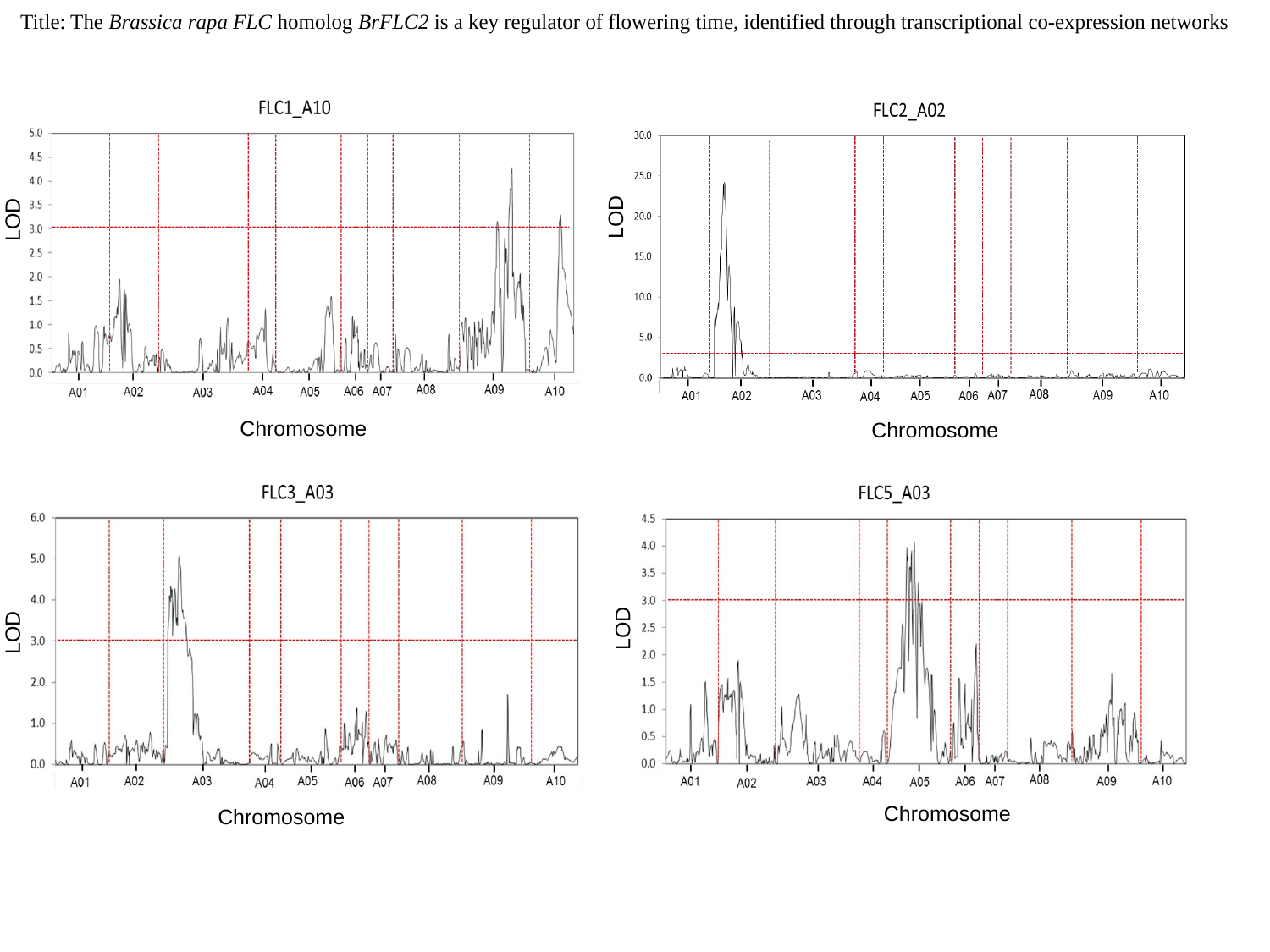

Title: The Brassica rapa FLC homolog BrFLC2 is a key regulator of flowering time, identified through transcriptional co-expression networks
LOD
Chromosome
Chromosome
LOD
LOD
Chromosome
LOD
Chromosome

## Slide 14
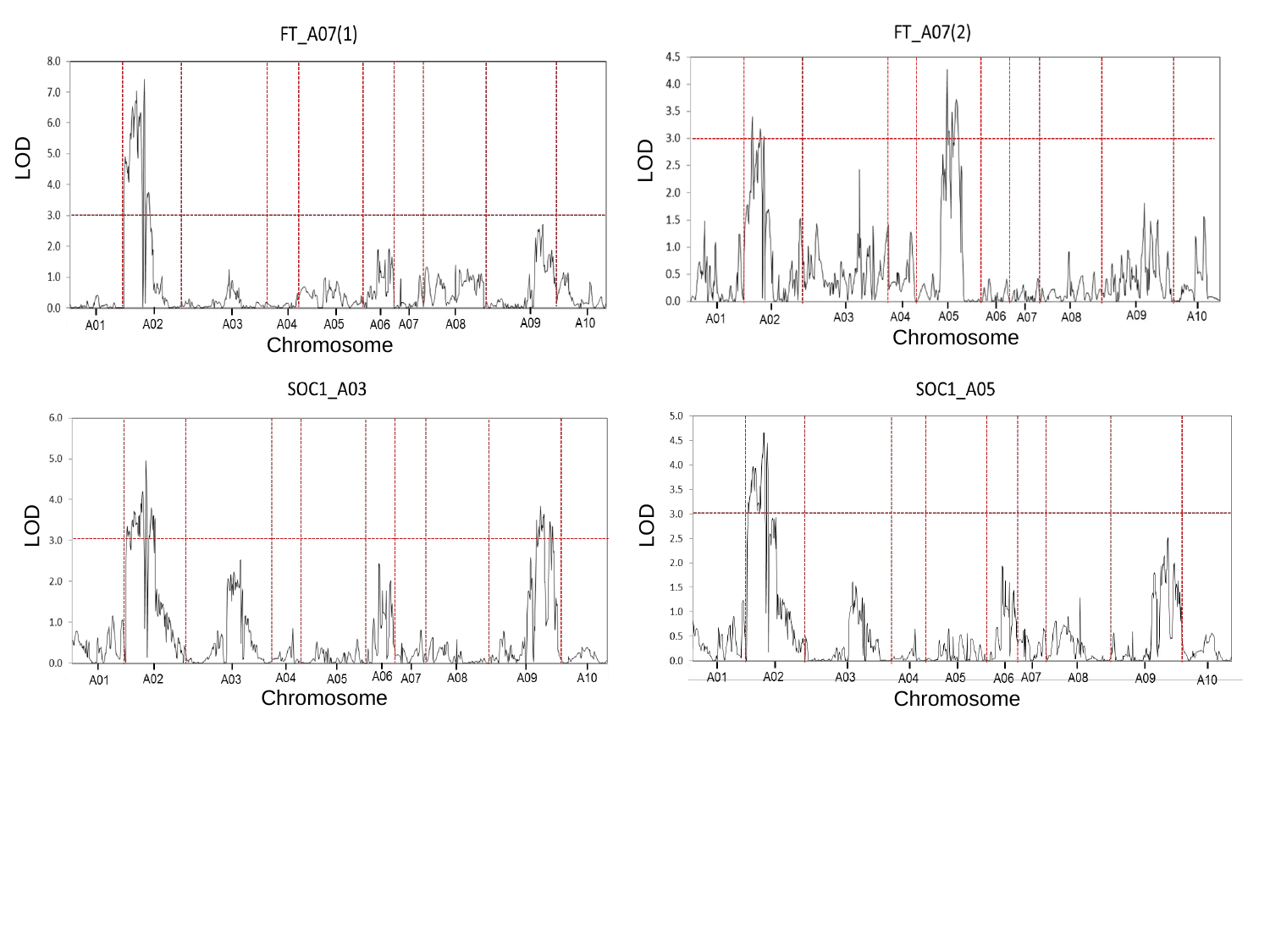

LOD
Chromosome
LOD
Chromosome
LOD
Chromosome
LOD
Chromosome

## Slide 15
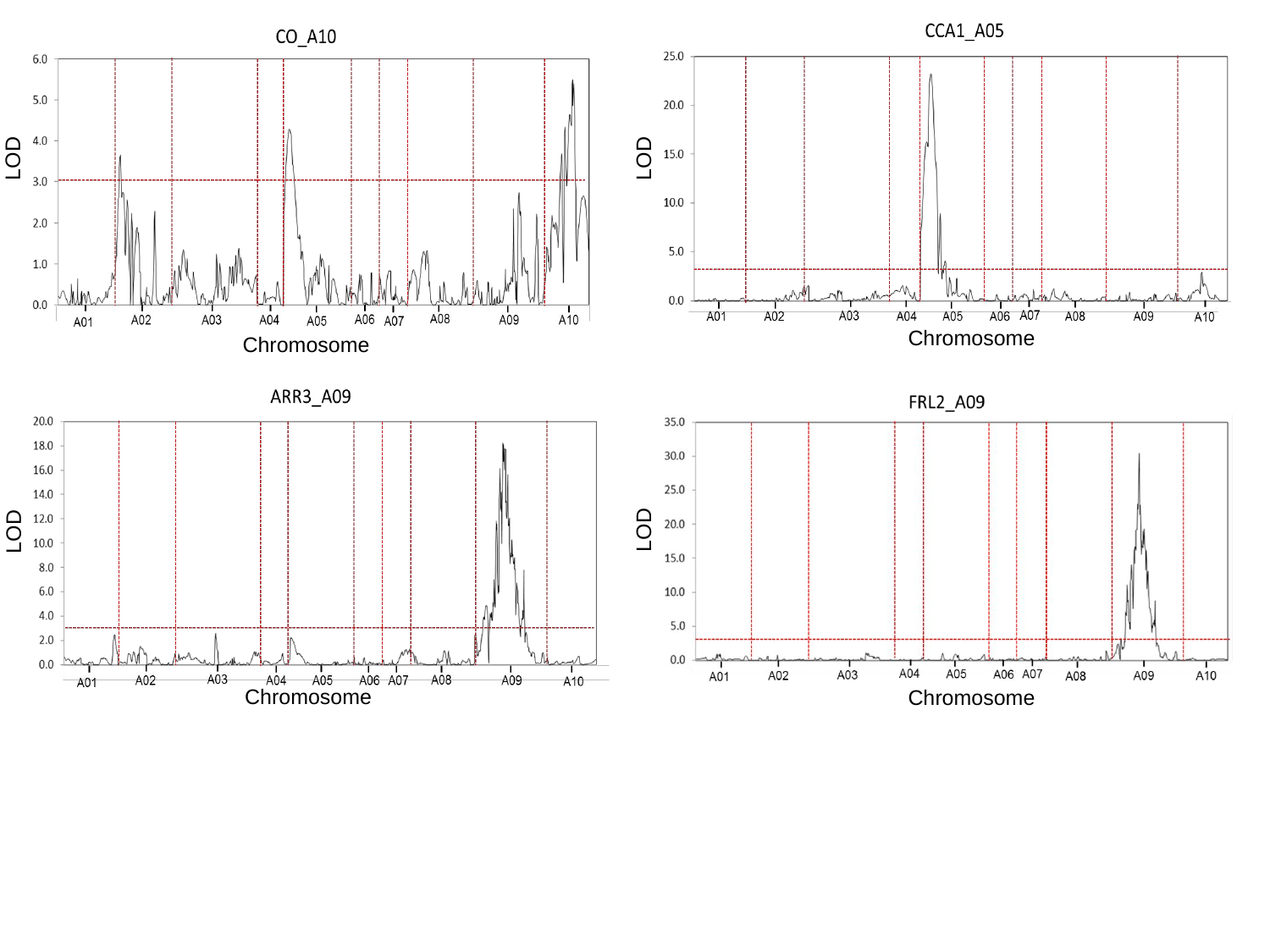

LOD
Chromosome
LOD
Chromosome
LOD
Chromosome
LOD
Chromosome

## Slide 16
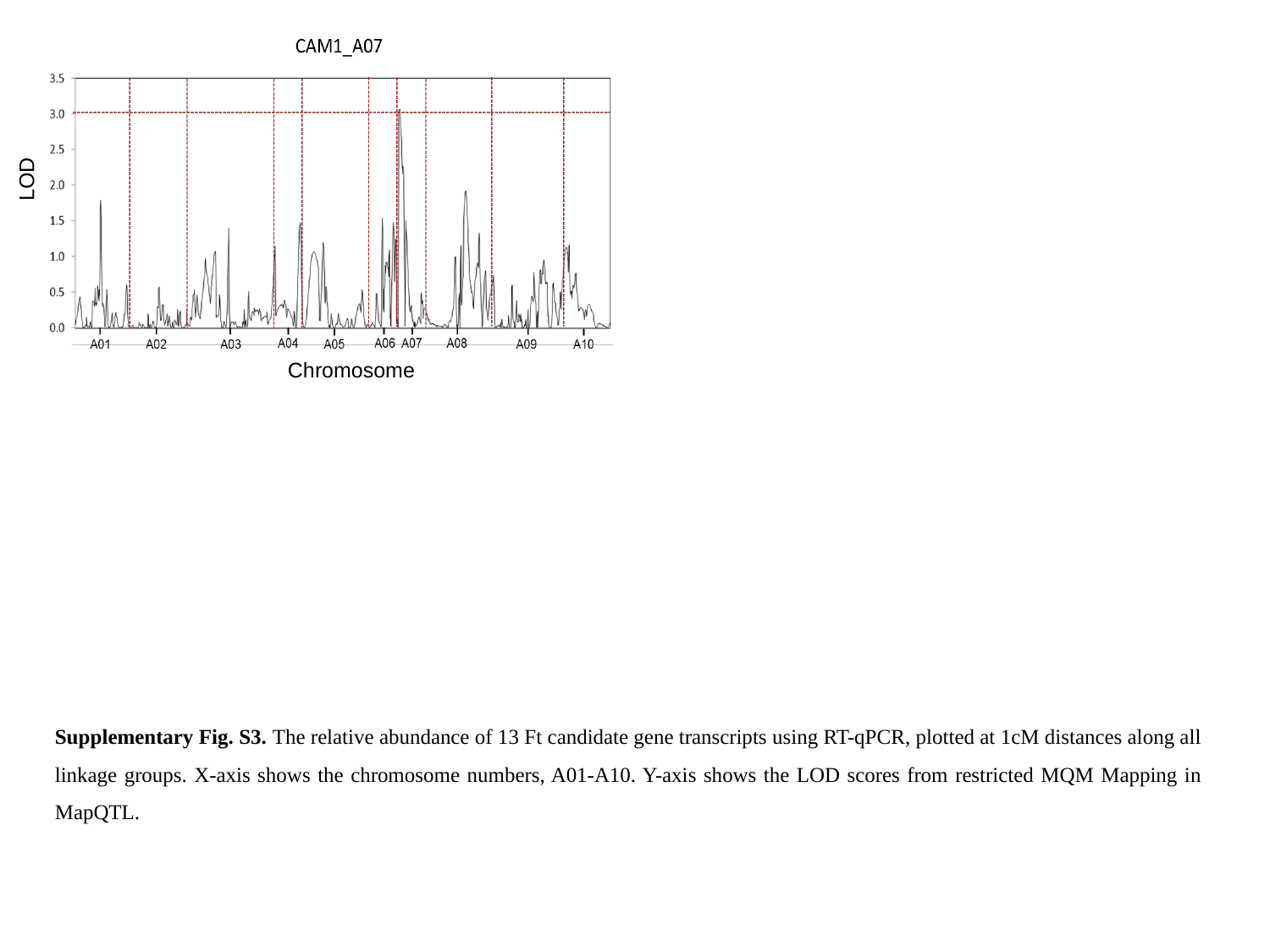

LOD
Chromosome
Supplementary Fig. S3. The relative abundance of 13 Ft candidate gene transcripts using RT-qPCR, plotted at 1cM distances along all linkage groups. X-axis shows the chromosome numbers, A01-A10. Y-axis shows the LOD scores from restricted MQM Mapping in MapQTL.

## Slide 17
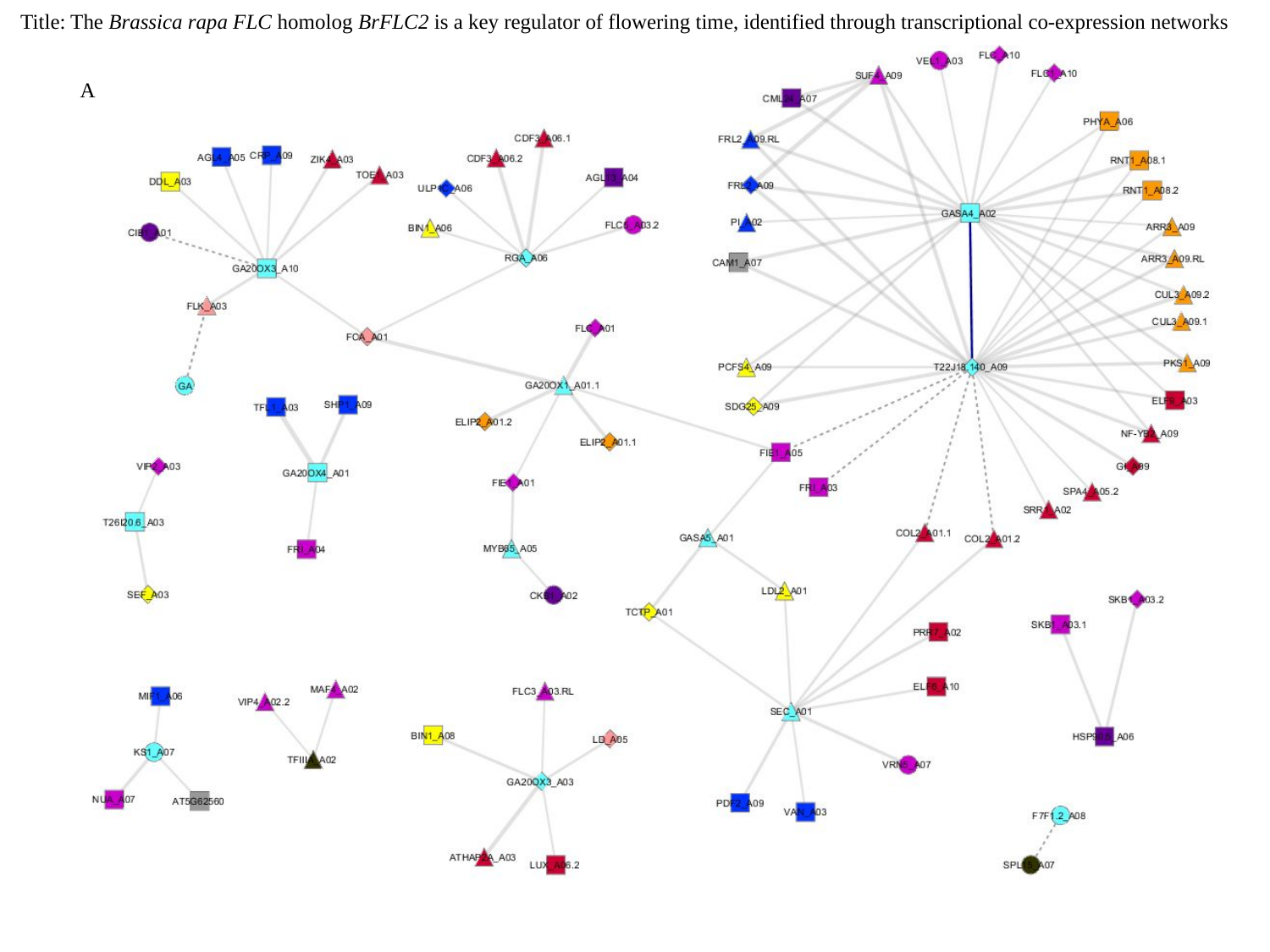

Title: The Brassica rapa FLC homolog BrFLC2 is a key regulator of flowering time, identified through transcriptional co-expression networks
A

## Slide 18
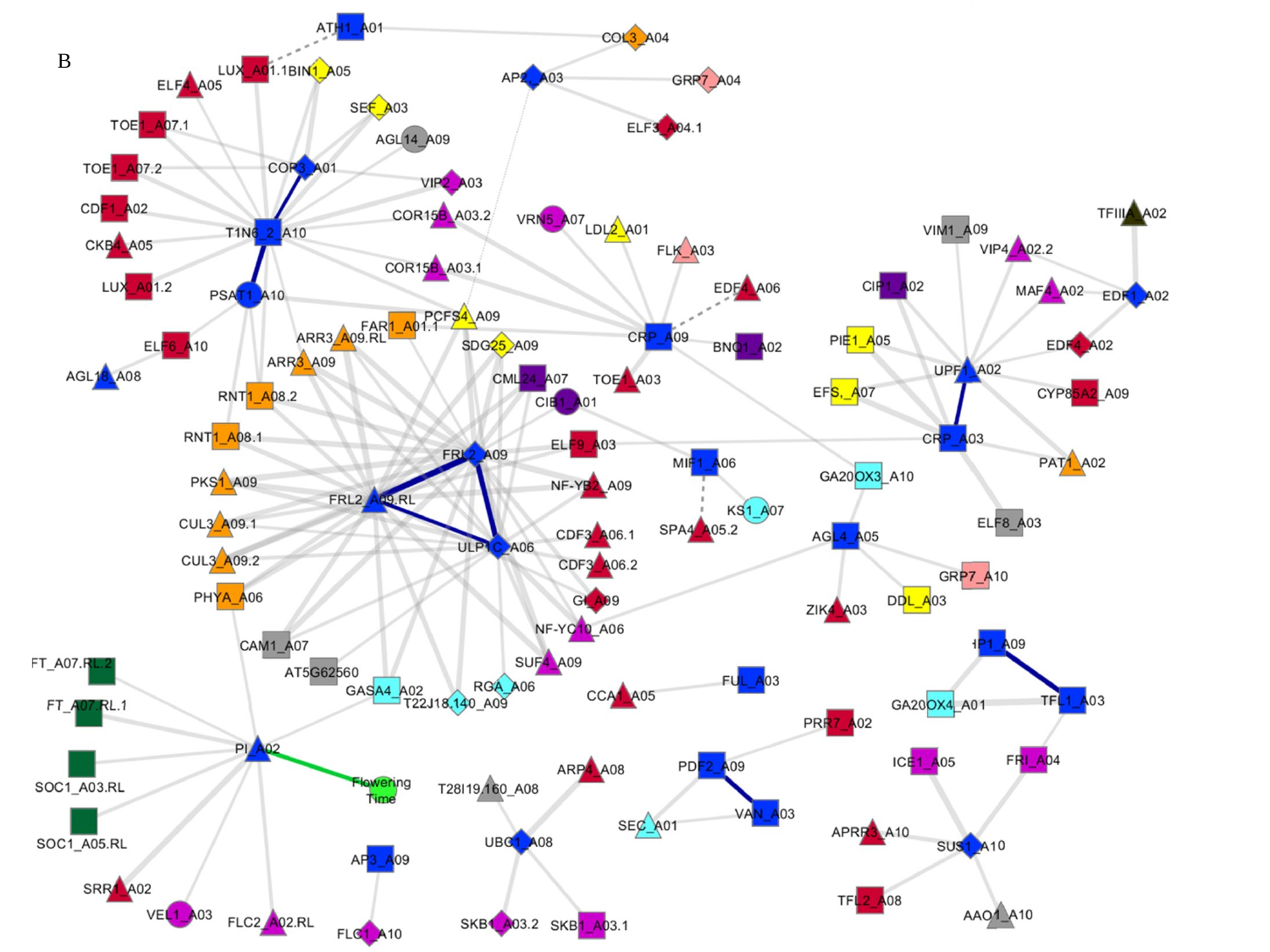

B

## Slide 19
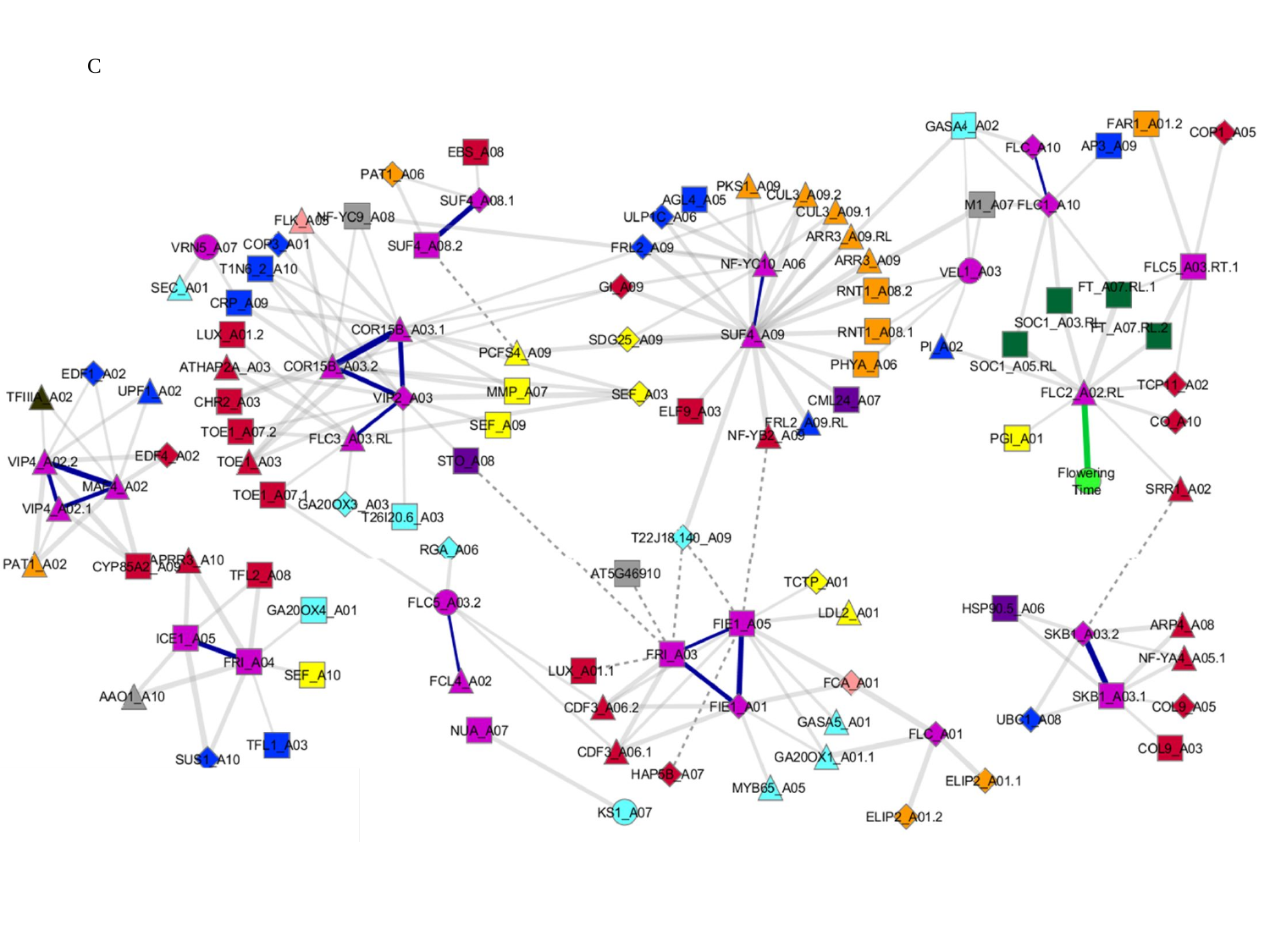

C

## Slide 20
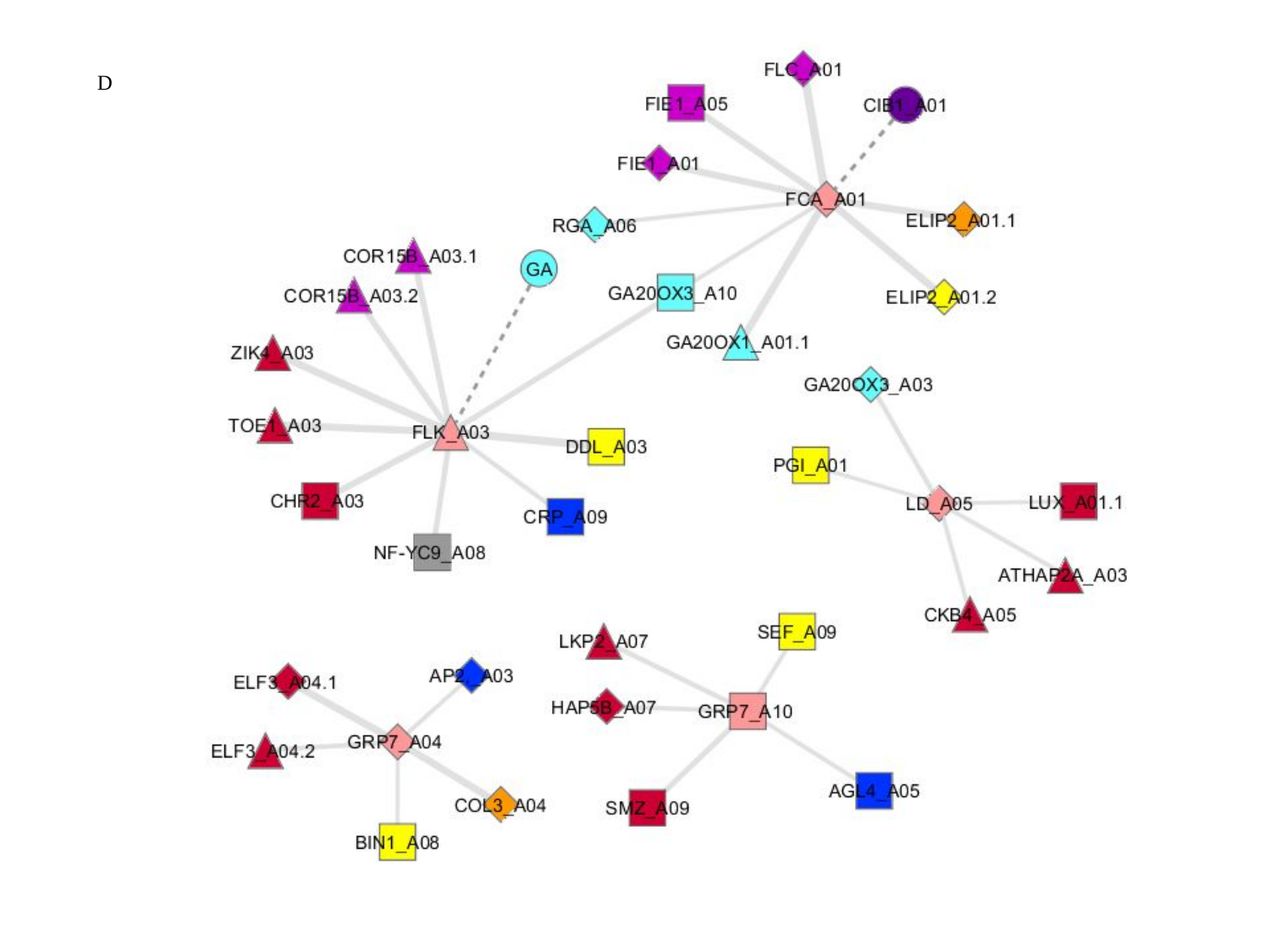

D

## Slide 21
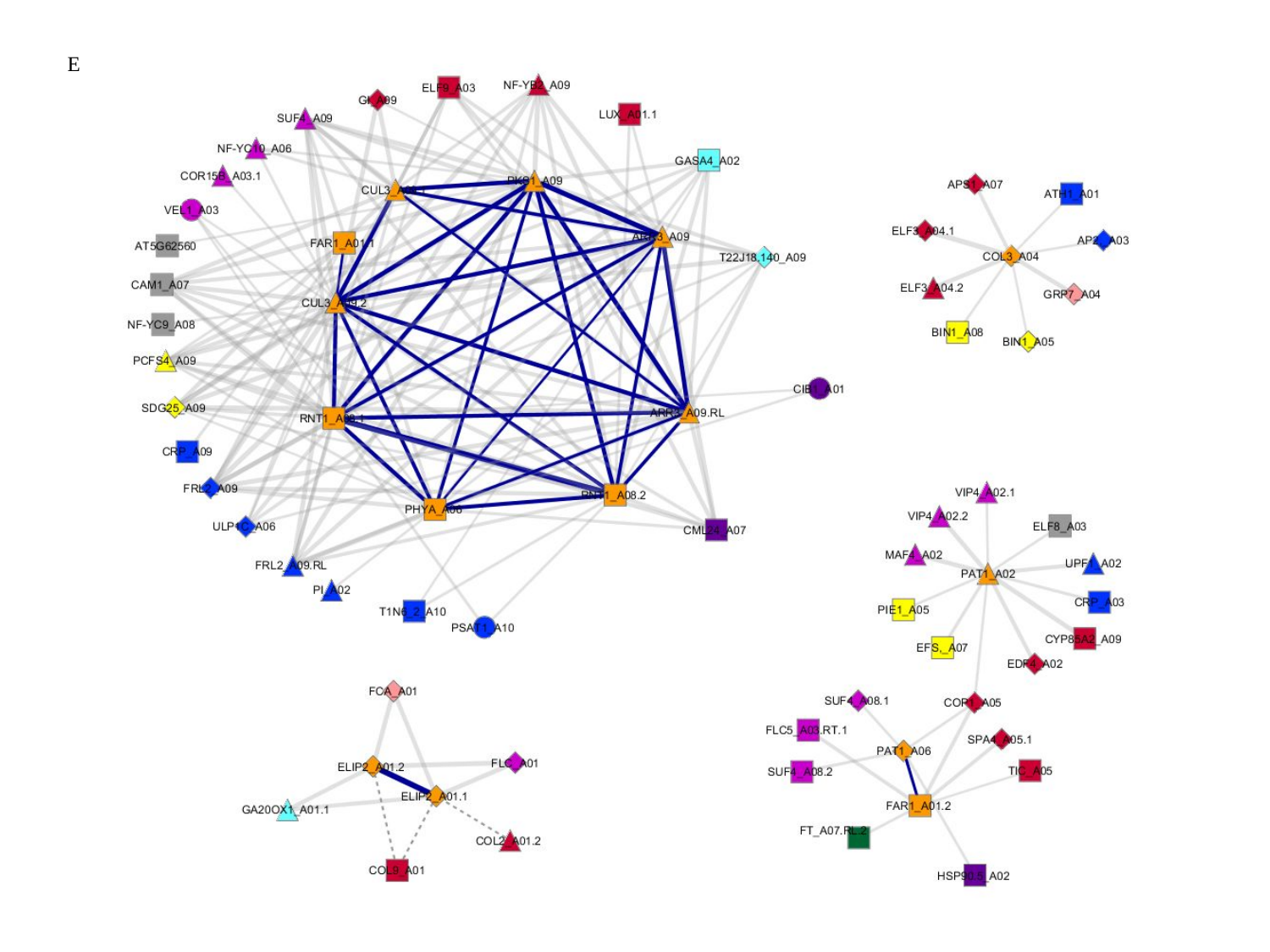

E

## Slide 22
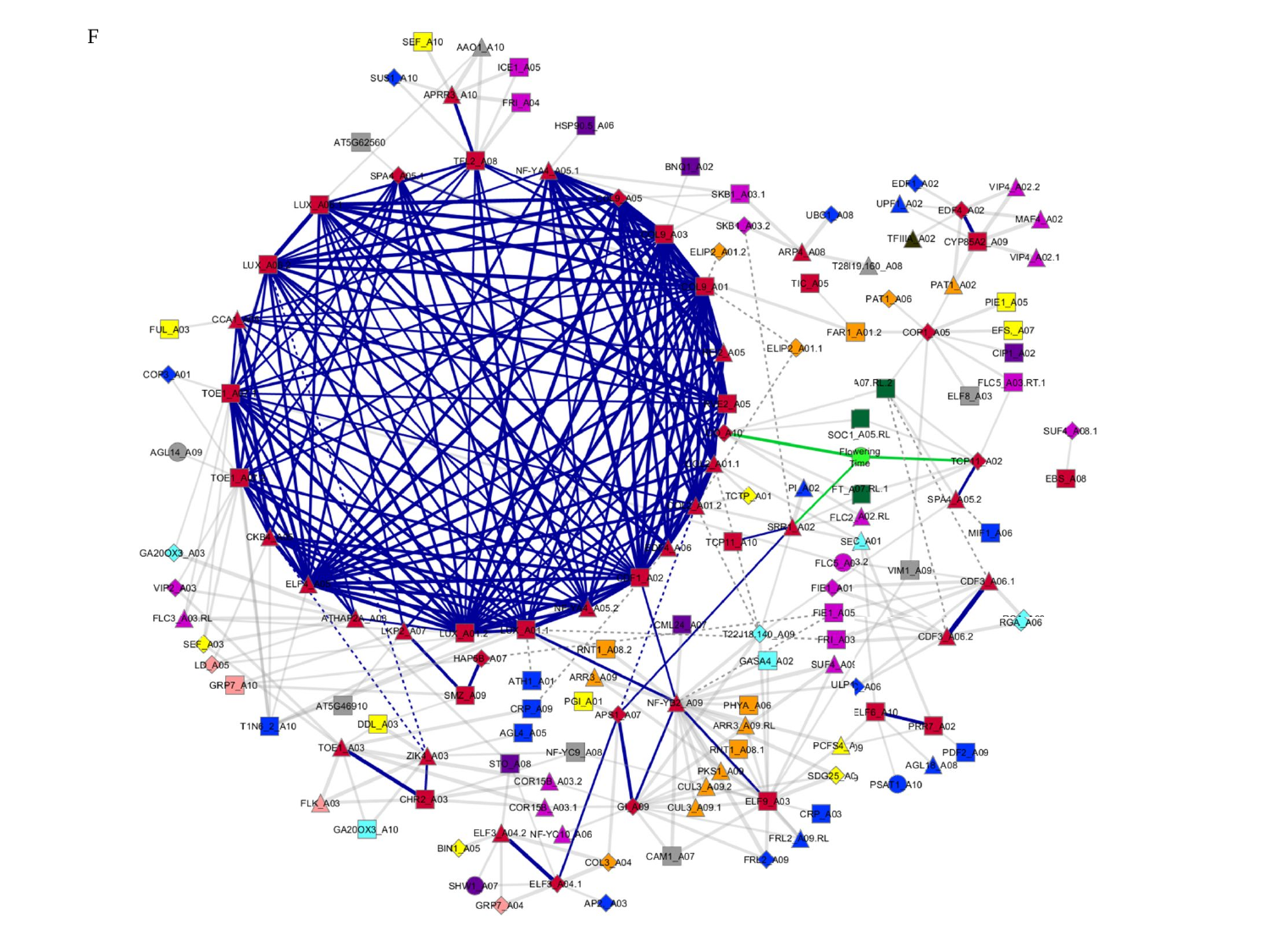

F

## Slide 23
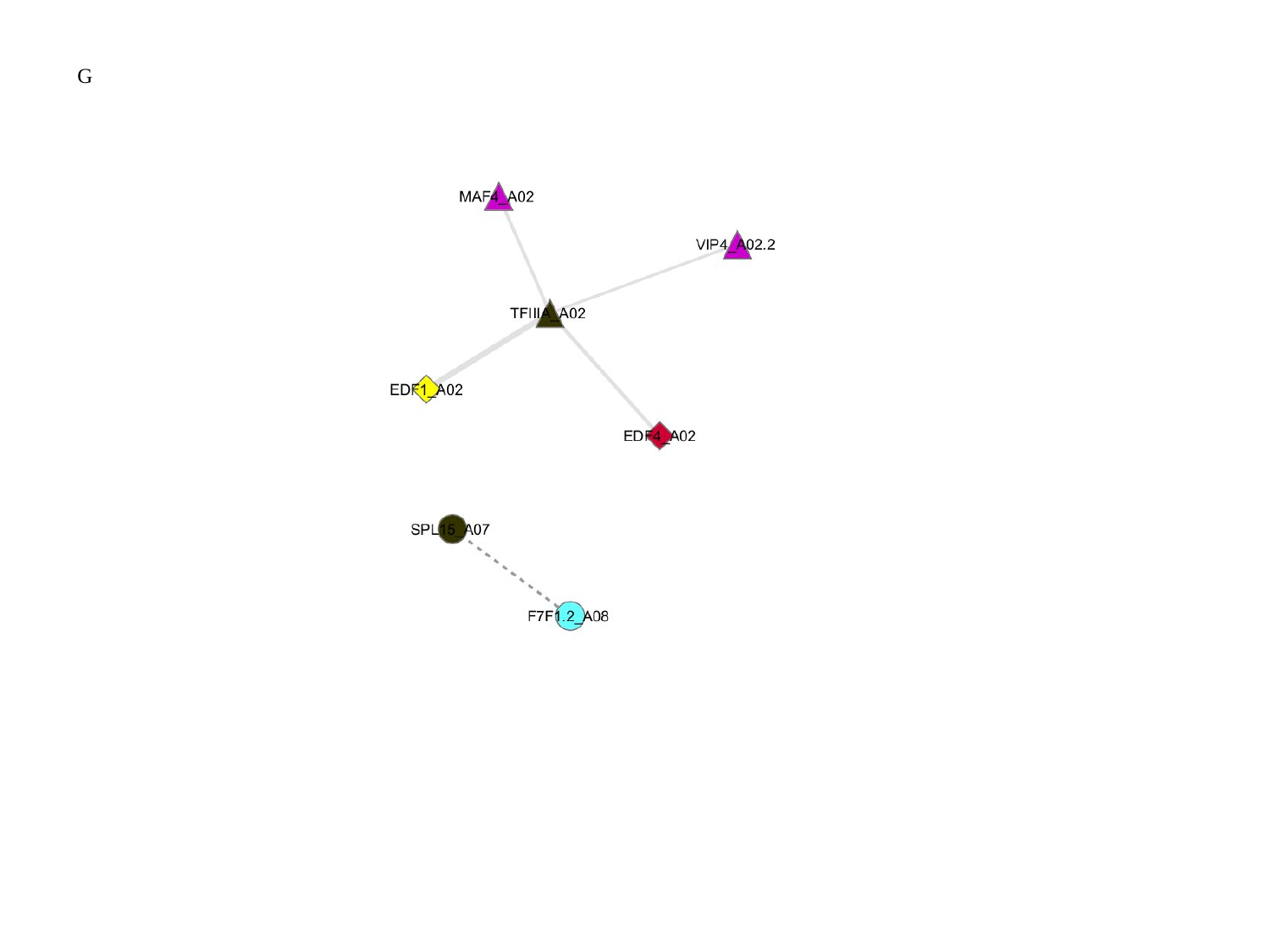

G

## Slide 24
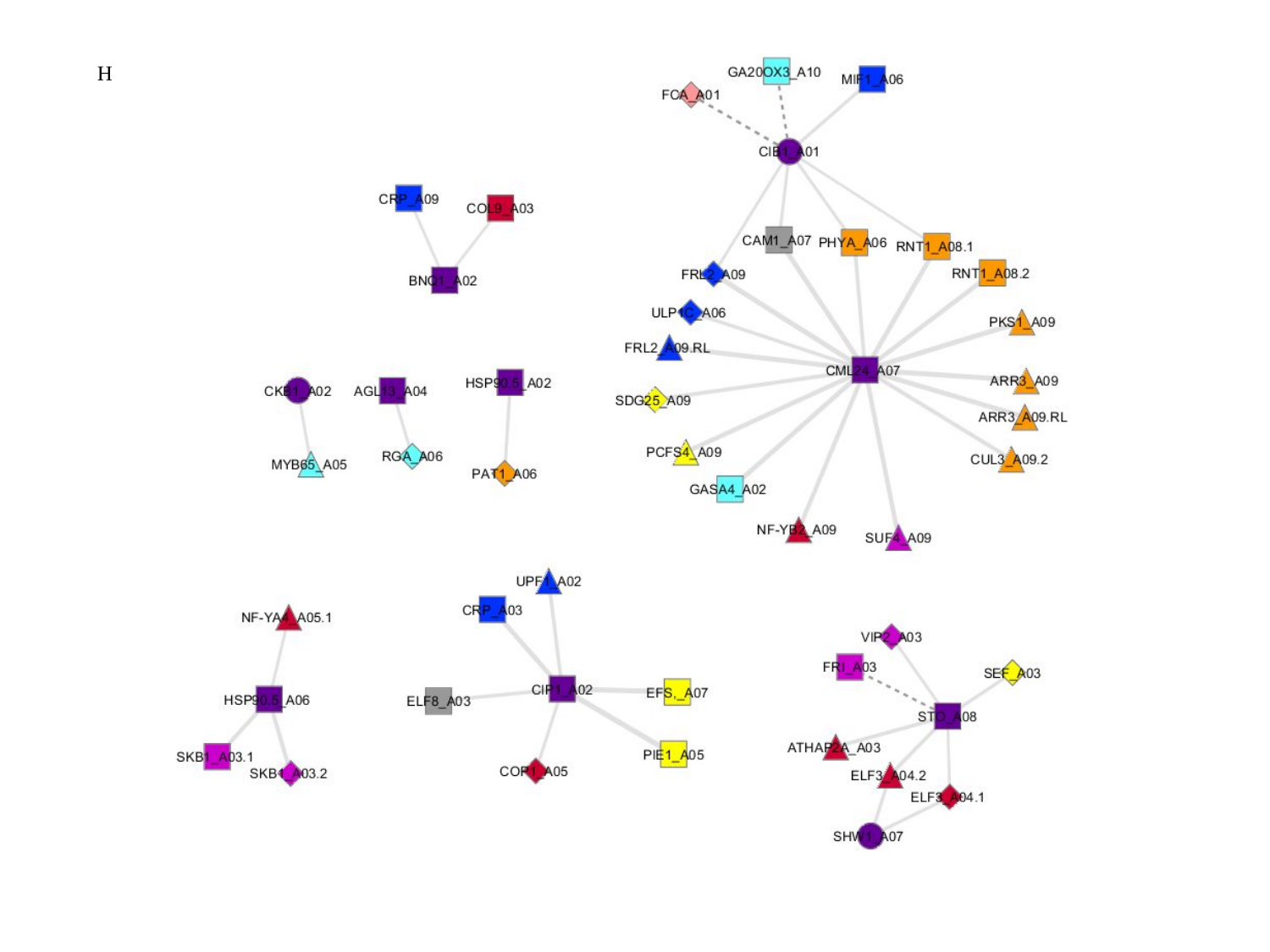

H

## Slide 25
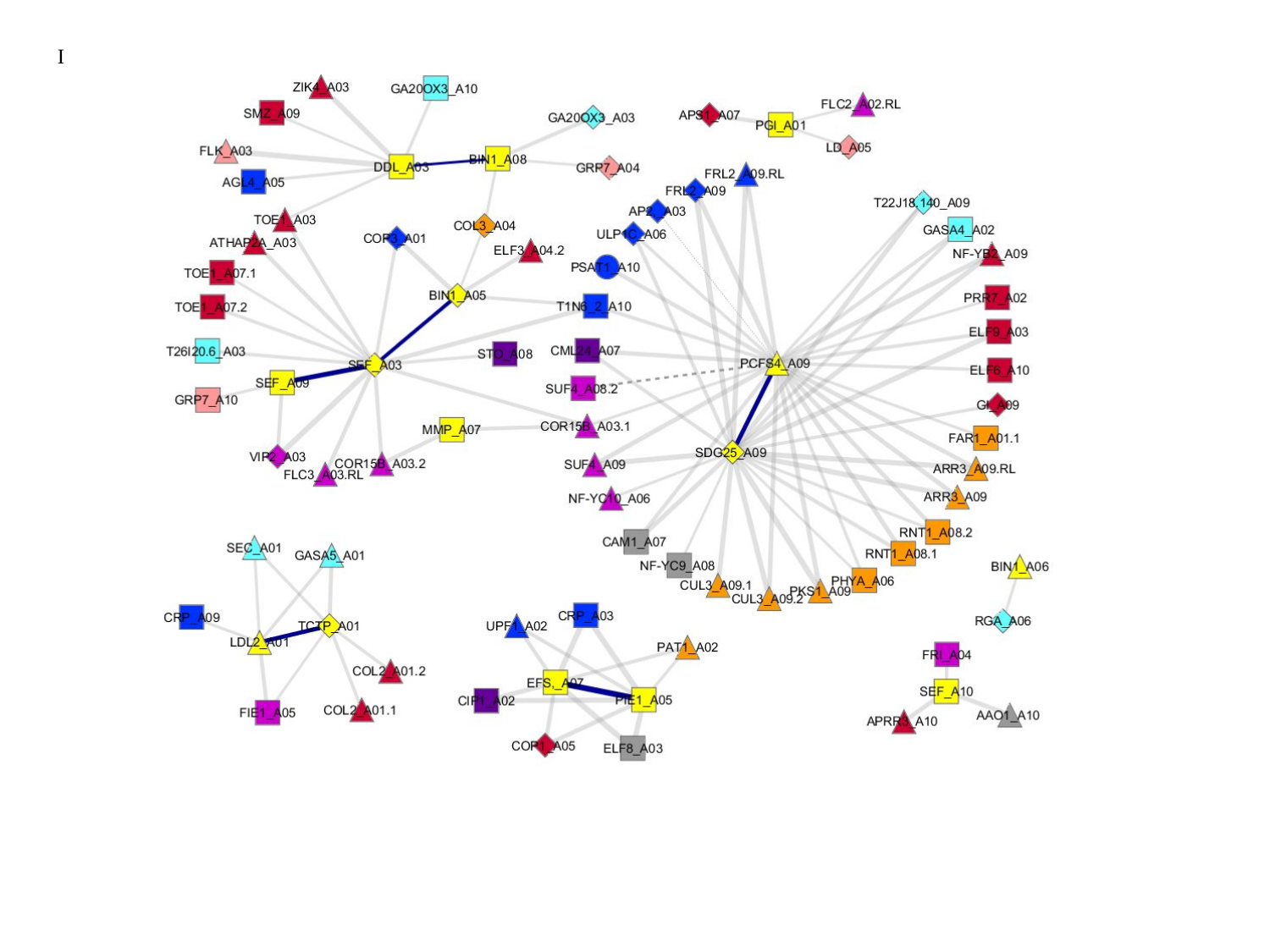

I

## Slide 26
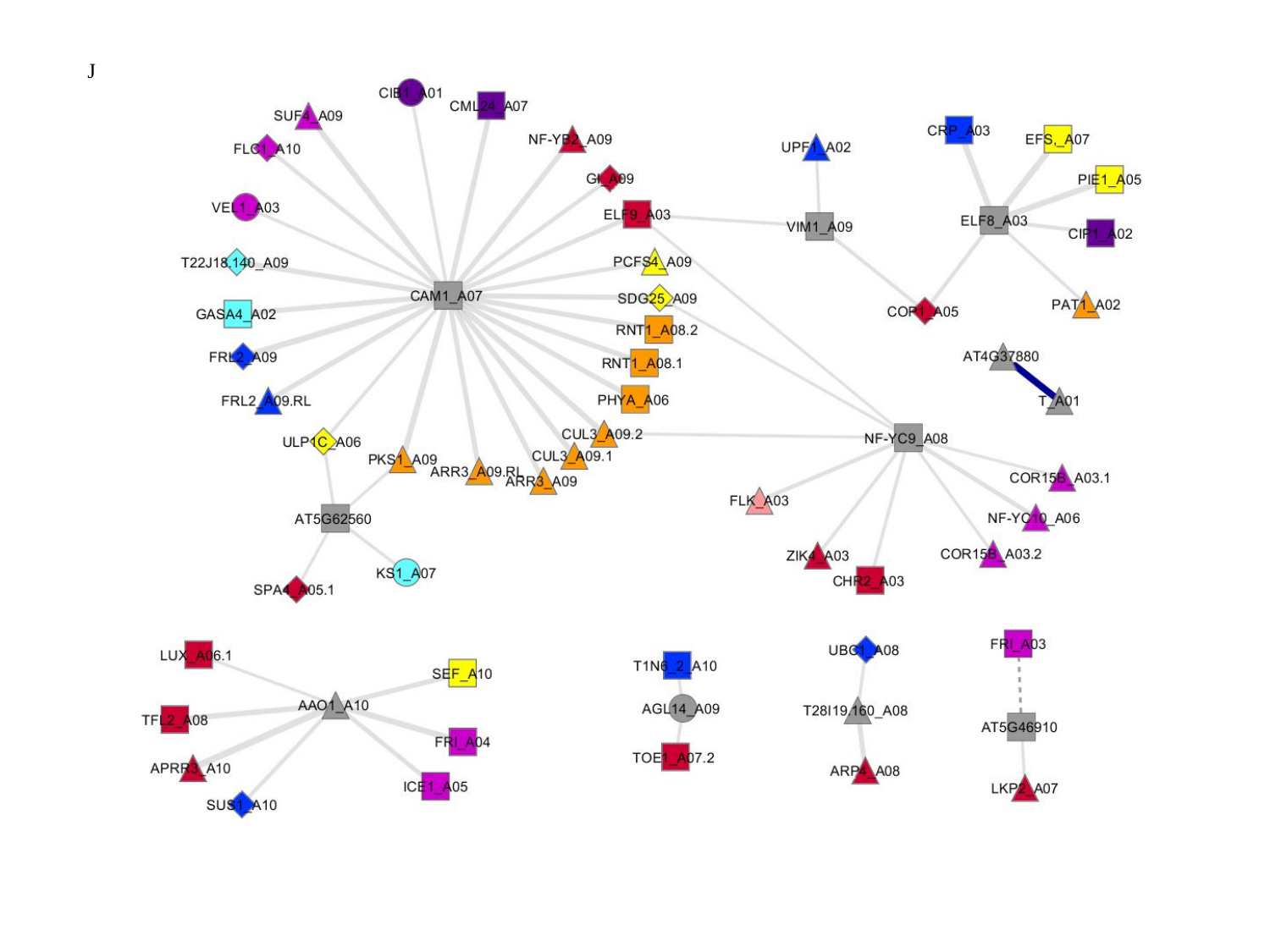

J

## Slide 27
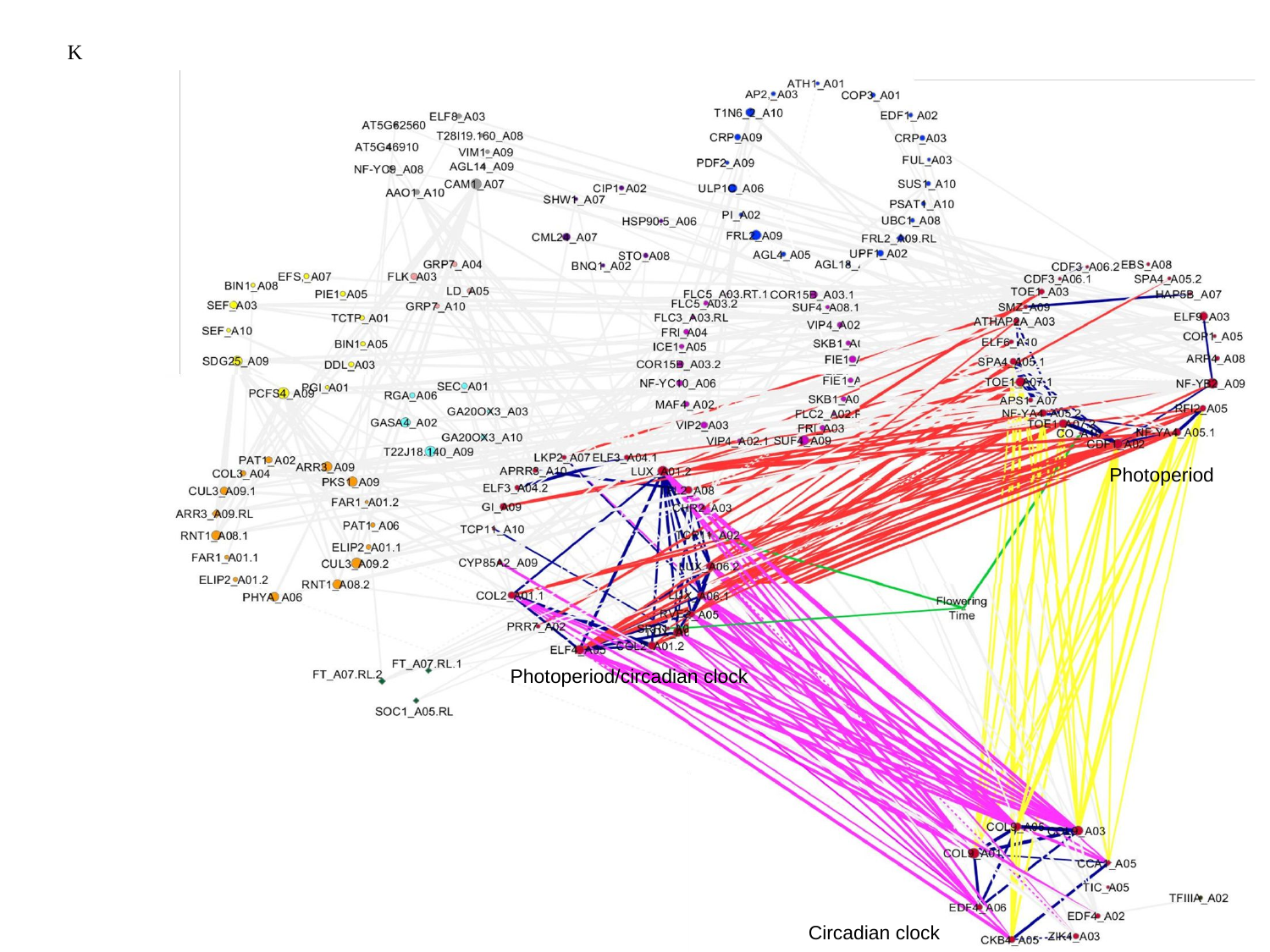

K
Photoperiod
Photoperiod/circadian clock
Circadian clock

## Slide 28
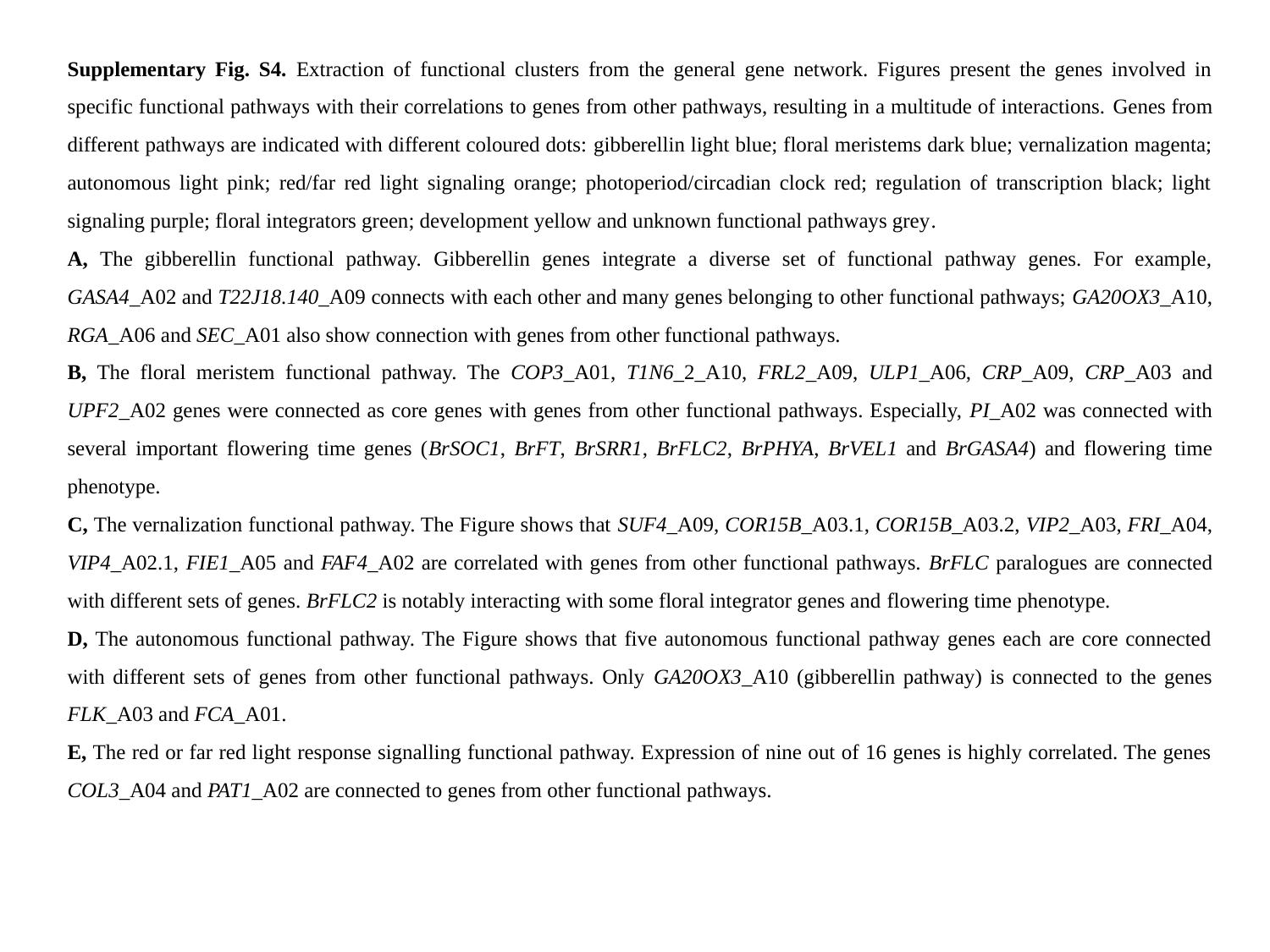

Supplementary Fig. S4. Extraction of functional clusters from the general gene network. Figures present the genes involved in specific functional pathways with their correlations to genes from other pathways, resulting in a multitude of interactions. Genes from different pathways are indicated with different coloured dots: gibberellin light blue; floral meristems dark blue; vernalization magenta; autonomous light pink; red/far red light signaling orange; photoperiod/circadian clock red; regulation of transcription black; light signaling purple; floral integrators green; development yellow and unknown functional pathways grey.
A, The gibberellin functional pathway. Gibberellin genes integrate a diverse set of functional pathway genes. For example, GASA4_A02 and T22J18.140_A09 connects with each other and many genes belonging to other functional pathways; GA20OX3_A10, RGA_A06 and SEC_A01 also show connection with genes from other functional pathways.
B, The floral meristem functional pathway. The COP3_A01, T1N6_2_A10, FRL2_A09, ULP1_A06, CRP_A09, CRP_A03 and UPF2_A02 genes were connected as core genes with genes from other functional pathways. Especially, PI_A02 was connected with several important flowering time genes (BrSOC1, BrFT, BrSRR1, BrFLC2, BrPHYA, BrVEL1 and BrGASA4) and flowering time phenotype.
C, The vernalization functional pathway. The Figure shows that SUF4_A09, COR15B_A03.1, COR15B_A03.2, VIP2_A03, FRI_A04, VIP4_A02.1, FIE1_A05 and FAF4_A02 are correlated with genes from other functional pathways. BrFLC paralogues are connected with different sets of genes. BrFLC2 is notably interacting with some floral integrator genes and flowering time phenotype.
D, The autonomous functional pathway. The Figure shows that five autonomous functional pathway genes each are core connected with different sets of genes from other functional pathways. Only GA20OX3_A10 (gibberellin pathway) is connected to the genes FLK_A03 and FCA_A01.
E, The red or far red light response signalling functional pathway. Expression of nine out of 16 genes is highly correlated. The genes COL3_A04 and PAT1_A02 are connected to genes from other functional pathways.

## Slide 29
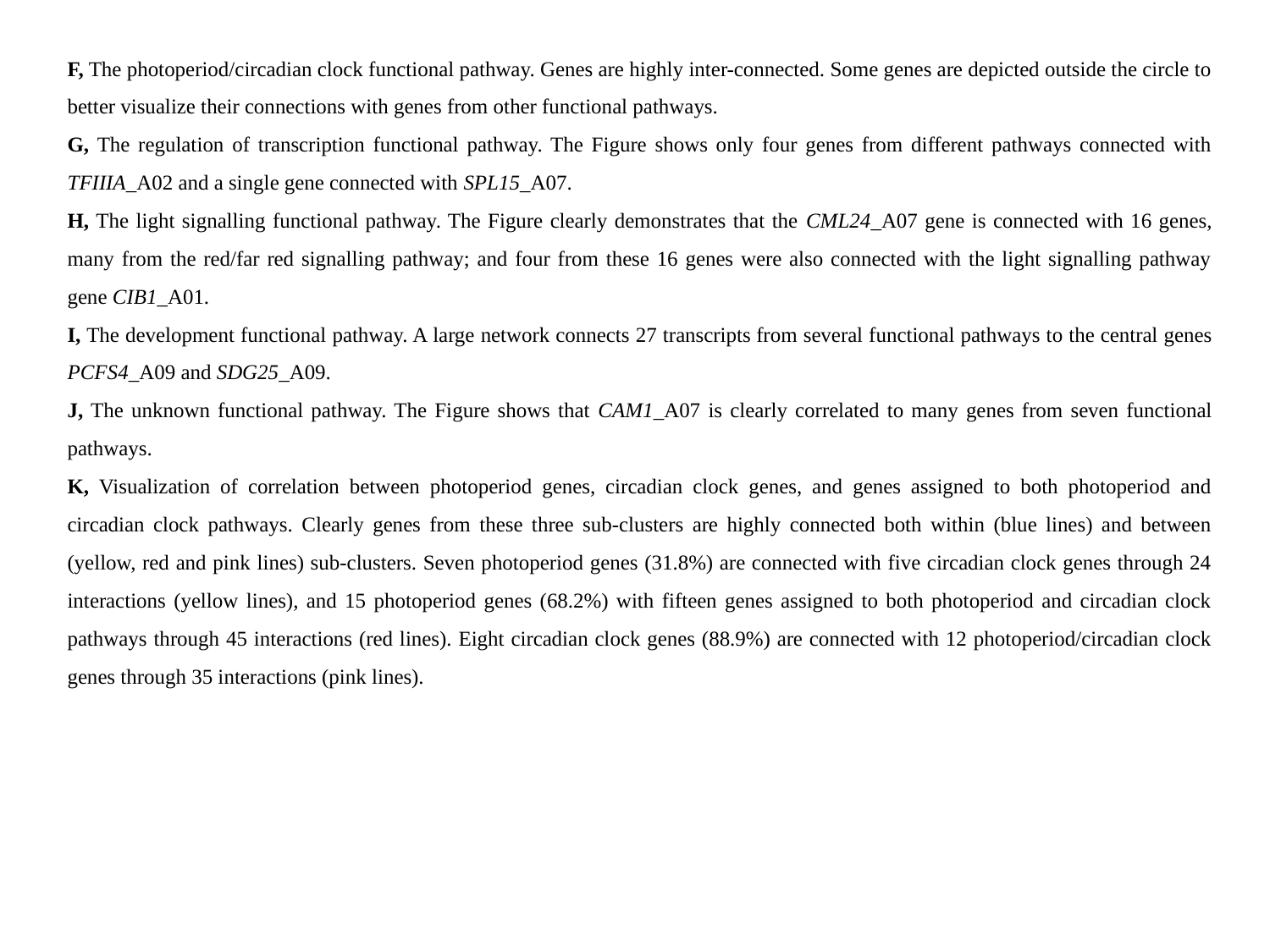

F, The photoperiod/circadian clock functional pathway. Genes are highly inter-connected. Some genes are depicted outside the circle to better visualize their connections with genes from other functional pathways.
G, The regulation of transcription functional pathway. The Figure shows only four genes from different pathways connected with TFIIIA_A02 and a single gene connected with SPL15_A07.
H, The light signalling functional pathway. The Figure clearly demonstrates that the CML24_A07 gene is connected with 16 genes, many from the red/far red signalling pathway; and four from these 16 genes were also connected with the light signalling pathway gene CIB1_A01.
I, The development functional pathway. A large network connects 27 transcripts from several functional pathways to the central genes PCFS4_A09 and SDG25_A09.
J, The unknown functional pathway. The Figure shows that CAM1_A07 is clearly correlated to many genes from seven functional pathways.
K, Visualization of correlation between photoperiod genes, circadian clock genes, and genes assigned to both photoperiod and circadian clock pathways. Clearly genes from these three sub-clusters are highly connected both within (blue lines) and between (yellow, red and pink lines) sub-clusters. Seven photoperiod genes (31.8%) are connected with five circadian clock genes through 24 interactions (yellow lines), and 15 photoperiod genes (68.2%) with fifteen genes assigned to both photoperiod and circadian clock pathways through 45 interactions (red lines). Eight circadian clock genes (88.9%) are connected with 12 photoperiod/circadian clock genes through 35 interactions (pink lines).

## Slide 30
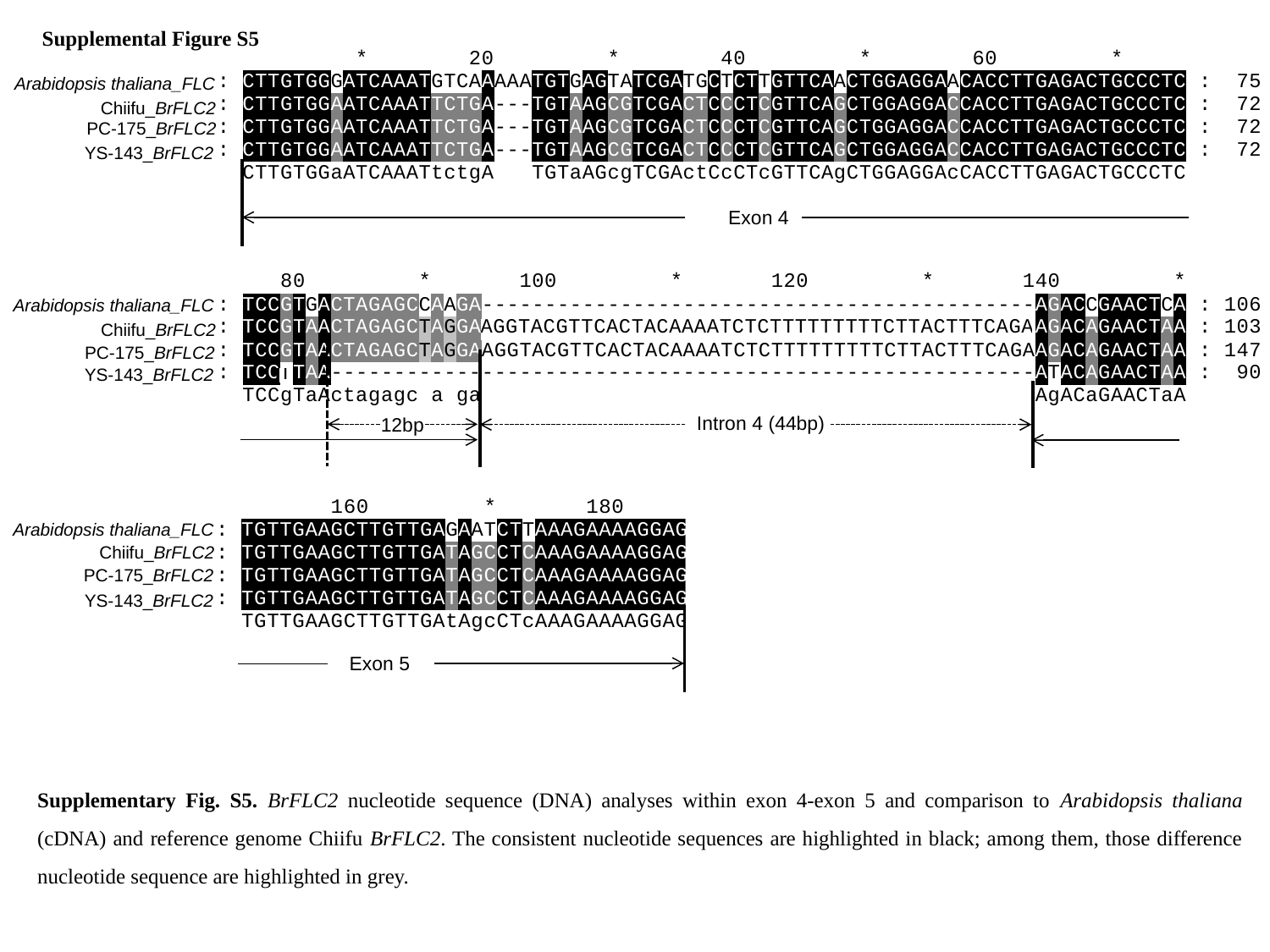

Supplemental Figure S5
Arabidopsis thaliana_FLC
Chiifu_BrFLC2
PC-175_BrFLC2
YS-143_BrFLC2
Exon 4
Arabidopsis thaliana_FLC
Chiifu_BrFLC2
PC-175_BrFLC2
YS-143_BrFLC2
Intron 4 (44bp)
12bp
Exon 5
Arabidopsis thaliana_FLC
Chiifu_BrFLC2
PC-175_BrFLC2
YS-143_BrFLC2
Supplementary Fig. S5. BrFLC2 nucleotide sequence (DNA) analyses within exon 4-exon 5 and comparison to Arabidopsis thaliana (cDNA) and reference genome Chiifu BrFLC2. The consistent nucleotide sequences are highlighted in black; among them, those difference nucleotide sequence are highlighted in grey.
